# Poor shoot and leaf growth in Huanglongbing-affected sweet orange is associated with increased investment in defenses

**DOI:** 10.3389/fpls.2023.1305815

**Published:** 2023-12-19

**Authors:** Answiya Neupane, Faisal Shahzad, Chiara Bernardini, Amit Levy, Tripti Vashisth

**Affiliations:** ^1^ Citrus Research and Education Center, University of Florida, Lake Alfred, FL, United States; ^2^ Department of Plant Pathology, University of Florida, Gainesville, FL, United States

**Keywords:** citrus, leaf growth, Huanglongbing, hormones, canopy

## Abstract

Citrus disease Huanglongbing (HLB) causes sparse (thinner) canopies due to reduced leaf and shoot biomass. Herein, we present results demonstrating the possible mechanisms behind compromised leaf growth of HLB-affected ‘Valencia’ sweet orange trees by comparing morphological, transcriptome, and phytohormone profiles at different leaf development phases (1. buds at the start of the experiment; 2. buds on day 5; . 3. leaf emergence; 4. leaf expansion; and 5. leaf maturation) to healthy trees. Over a period of 3 months (in greenhouse conditions), HLB-affected trees had ≈40% reduction in growth traits such as tree height, number of shoots per tree, shoot length, internode length, and leaf size compared to healthy trees. In addition, buds from HLB-affected trees lagged by ≈1 week in sprouting as well as leaf growth. Throughout the leaf development, high accumulation of defense hormones, salicylic acid (SA) and abscisic acid (ABA), and low levels of growth-promoting hormone (auxin) were found in HLB-affected trees compared to healthy trees. Concomitantly, HLB-affected trees had upregulated differentially expressed genes (DEGs) encoding SA, ABA, and ethylene-related proteins in comparison to healthy trees. The total number of cells per leaf was lower in HLB-affected trees compared to healthy trees, which suggests that reduced cell division may coincide with low levels of growth-promoting hormones leading to small leaf size. Both bud dieback and leaf drop were higher in HLB-affected trees than in healthy trees, with concomitant upregulated DEGs encoding senescence-related proteins in HLB-affected trees that possibly resulted in accelerated aging and cell death. Taken together, it can be concluded that HLB-affected trees had a higher tradeoff of resources on defense over growth, leading to sparse canopies and a high tree mortality rate with HLB progression.

## 1 Introduction

Huanglongbing (HLB), or citrus greening, disease has become the biggest challenge for citrus growers and researchers in sustaining the Florida citrus industry. HLB has caused an 80% citrus production decline over the past two decades (USDA, 2021). HLB is presumably caused by the bacterium *Candidatus* Liberibacter asiaticus (*C*Las), an uncultured, phloem-limited, gram-negative α-proteobacterium (Garnier and Bové, 1983; Jagoueix et al., 1994), which is transmitted by the two species of the citrus psyllid *Diaphorina citri* (Asian citrus psyllid (ACP)) and *Trioza erytreae* (African citrus psyllid) (Bové, 2006). To date, there is no cure for HLB, and no citrus germplasm show resistance against HLB. Following *C*Las infection, HLB-affected trees exhibit nutrient deficiencies, small leaves, blotchy leaf mottle, twig dieback, and stunted growth (Schneider, 1968; Li et al., 2003; Bové, 2006; Etxeberria et al., 2009; Shahzad et al., 2020). In addition, loss of feeder roots and phloem plugging via callose deposition (Koh et al., 2012) result in limited nutrients, water, and carbohydrate translocation within the tree body and ultimately a decline in fruit productivity and tree life span. In aboveground tree body, leaves are the first plant organs to exhibit HLB characteristic symptoms.

Leaves are the primary source of photosynthesis; fruit growth and production depend on the partitioning of assimilated carbon sources between photosynthetically active sources, mature leaves, and photosynthetically less active sink tissues such as fruit and roots (Baldet et al., 2002). In Florida, three cycles of vegetative flush occur throughout the year: one in February–March, followed by one in May–June, and one in August–September (O'Brien, 2016). The leaf development process begins with bud break, followed by flush development, leaf expansion, and finally maturation to full photosynthetic capacity (Cifuentes-Arenas et al., 2018). The life span of a leaf can vary from a few weeks to several years, with an average of 1.5 years. Senescent leaves are replaced by newly developing leaves, and the process goes on continuously (Ribeiro et al., 2021). However, HLB-affected trees exhibit arrested growth patterns, reduced leaf size, and accelerated leaf drop, resulting in sparse canopies and tree mortality (Keeley et al., 2022; Shahzad et al., 2023). The underlying mechanism behind HLB-triggered compromised leaf growth has not been discovered yet. Thus, the goal of this research was to understand the cause of differential leaf development in HLB-affected trees via morphological, transcriptome, and phytohormone analyses. This basic understanding will help in developing strategies to mitigate the negative effects of HLB on leaf growth.

## 2 Materials and methods

### 2.1 Plant materials

**Figure 1:**
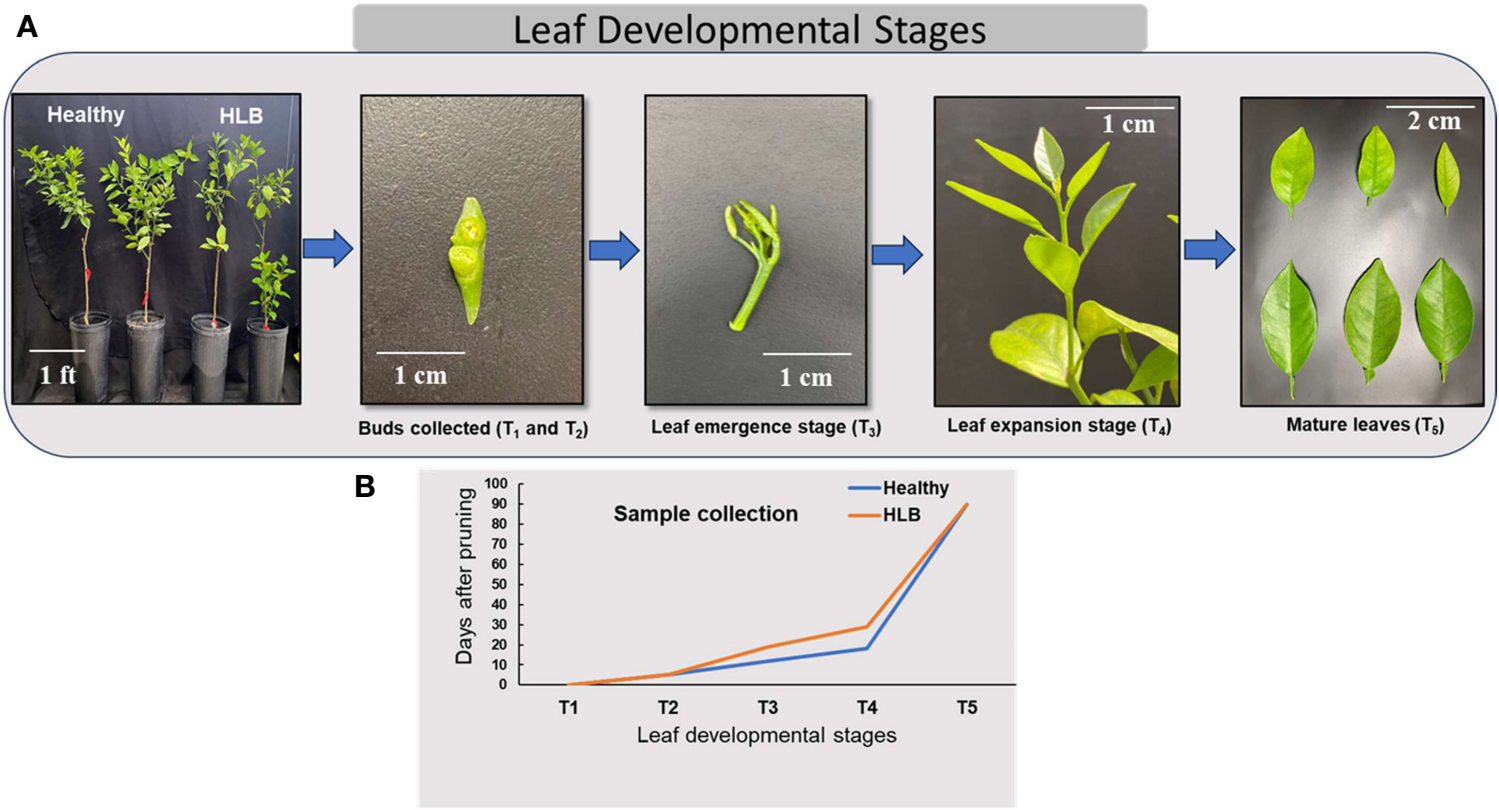
Stages of leaf development in citrus **(A)** and sample collection during different leaf development phases from healthy (HLY) and Huanglongbing (HLB)-affected trees **(B)**. T_1_, buds at the start of experiment; T_2_, buds on day 5; T_3_, leaf emergence; T_4_, leaf expansion; T_5_, leaf maturation.

Seven-year-old sweet orange ‘Valencia’ grafted on Swingle citrumelo rootstock (*Citrus paradisi* × *Poncirus trifoliata*) citrus trees potted (pot dimensions: 10.2 × 10.2 × 35.6 cm) in commercial citrus growing media (mixture of peat/perlite/vermiculite at 3:1:1 by volume) and grown in a greenhouse located at the Citrus Research and Education Center, Lake Alfred, Florida, were used in this study. For HLB positive, trees were inoculated with *C*Las-positive buds, and healthy (HLY) trees were mock-inoculated with healthy buds (referred to as disease conditions: HLY and HLB-affected trees). *C*Las infection was confirmed in all infected trees using quantitative real-time polymerase chain reaction (qPCR) as described in Vashisth and Livington (2019) and represented as cycle threshold (value of *C*Las presence in HLB-affected trees was 26). At the start of the experiment, all the trees were pruned to 40%–50% original canopy volume to force the trees to flush at the same time and allow for synchronized shoot development observations. Throughout the experiment, all the trees were kept in a temperature-controlled greenhouse with natural light, the temperature and relative humidity of the greenhouse fluctuated between 22°C and 25°C and 60% to 80%, respectively. The trees were fertilized regularly with a tap water mix of a water-soluble 20N–20P–20K plus micronutrient fertilizer. The experiment was carried out for 3 months (March–June) in 2022. The experiment was set up as a completely randomized design with eight individual tree replicates for two tree conditions, HLY or HLB-affected trees. Samples were collected at five different stages of leaf development: T_1_ = buds were collected on the day of pruning at the start of the experiment; T_2_ = buds at day 5; T_3_ = new flushes were collected at the leaf emergence stage; T_4_ = leaf expansion; and T_5_ = leaf maturation (**Figure 1A**). Bud samples for T_1_ and T_2_ were collected at the start of the experiment and after 5 days, respectively. For the T_3_ and T_4_ leaf stages, samples were collected at different times for both HLY and HLB-affected trees as the leaf emergence and growth were slow in HLB-affected trees (approximately 10 days) compared to HLY trees (**Figure 1B**). Regarding the T_5_ leaf stage, samples were collected at the fully matured stage at the end of the experiment (3 months old).

### 2.2 Physiological attributes

Tree growth attributes including tree height, trunk diameter, and SPAD value (chlorophyll index) were measured at an interval of 15 days for the course of the study. The number of sprouted buds and bud dieback were measured on a weekly basis until no bud growth was observed. Shoot length, internode length, number of leaves per shoot, leaf size, and leaf weight were measured for the new flushes that grew after pruning until the end of the study. Tree height was measured using a metric ruler (cm) from the media surface to the tip of the tree. Trunk diameter (cm) was measured using a vernier caliper 1 cm above the tree graft union. Leaf chlorophyll index (SPAD value) was measured for five same-age leaves using a chlorophyll concentration meter (MC-100; Apogee Instruments, Logan, UT, USA). The number of sprouted buds, leaves per shoot, leaf drop, and dead buds were counted manually. Leaves were weighed using a digital weighing balance. Samples (shoot length, internode length, and leaf size) were scanned at 24 DPI using an Epson Perfection V37 flatbed scanner (Epson America, Inc., Long Beach, CA, USA) using a dark blue background. Image analysis was performed using ImageJ software (National Institutes of Health, Bethesda, MD, USA; Schindelin et al., 2012).

### 2.3 Anatomical analysis

For anatomical analysis, three leaves per replicate were collected from the T_5_ stage (3-month-old mature leaves). After detachment, leaf sections were taken from the tip and bottom portions and immersed overnight in a solution containing 85% ethanol and 15% deionized water to remove chlorophyll. A simple microscope was used for capturing images using the Leica Confocal Software (Leica Microsystems GmbH, Mannheim, Germany). For counting and measuring cell sizes, image analysis was performed on a space measuring 100 * 75 μm^2^ using ImageJ software (National Institutes of Health, Bethesda, MD, USA; Schindelin et al., 2012).

### 2.4 Transcriptome analysis

For transcriptome analysis, leaf samples were collected at two stages: T_2_ = buds at day 5 and T_3_ = leaf emergence stage. RNA was extracted from 100-mg tissue using the RNeasy Mini Plant RNA Extraction Kit (Qiagen, Valencia, CA, USA). The quality and quantity of the extracted RNA were evaluated using a spectrophotometer (Epoch 2 Microplate: BioTek Instruments, Winooski, VT, USA) and denaturing formaldehyde 1.2% agarose gels (Rio, 2015). Subsequently, RNA samples of both HLY and HLB-affected ‘Valencia’ sweet orange (n = 4) at both leaf stages (T_2_ and T_3_) were sent to the University of Florida’s Interdisciplinary Center for Biotechnology Research (ICBR, UF) for global transcriptome analysis using Illumina RNA sequencing (RNA-seq). The RNA-seq raw reads are available at the National Center for Biotechnology Information (NCBI) Sequence Read Archive (SRA) database (https://www.ncbi.nlm.nih.gov/bioproject/) under accession number PRJNA1042812.

#### 2.4.1 Mapping of the reads, transcript count, and DEG analysis

Raw reads obtained from leaf samples were aligned with the genome of *Citrus sinensis* (version 154_v1.1) from JGI (Joint Genome Institute, Berkeley, CA, USA) for RNA-seq analysis using the read mapper of the STAR package (Spliced Transcripts Alignment to a Reference, v2.7.9a) (Dobin et al., 2013). The mapping results were further processed using the HTSeq (High-Throughput Sequence Analysis in Python, v0.11.2) (Anders et al., 2015), Sam tools, and scripts developed in-house at ICBR, UF, to remove potential PCR duplicates and choose and count uniquely mapped reads for gene expression analysis. Principal component analysis (PCA) (for detecting outlier samples) based on all identified genes in each analysis was performed using the R-package (v4.1.3). The counted reads of each gene were analyzed using a DESeq2-based R pipeline. Significant up- and downregulated genes were selected using the p-value (<0.05) and log_2_ fold-change (>1) for downstream analysis.

#### 2.4.2 Gene ontology enrichment and pathway analysis

Several tools were used to study the biological significance of the results of RNA-seq. MapMan software (version 3.5.1.R2) (Thimm et al., 2004) was used to identify the physiological or biochemical processes represented by the differentially expressed genes (DEGs); the percentage of the number of DEGs in each major functional category (BIN) over the total DEG number was calculated. For the gene ontology (GO) enrichment analysis, AgriGO was used in tandem with REViGO (Supek et al., 2011) to obtain statistically significant (*p* < 0.05) and non-redundant GO terms for upregulated and downregulated DEGs, respectively, in HLB-affected trees compared to HLY trees. Subsequently, GO terms were ranked by the degree of enrichment based on their enrichment scores (ESs); for each GO term, ES was calculated as follows: ES = (DEG number in the GO term/total DEG number)/(gene number in the genome for this GO term/total gene number in the genome).

qPCR was used to validate the results obtained from RNA-seq following the steps described by Tang and Vashisth (2020), and gene-specific primer sequences are listed in **Supplementary Table S1**. Eight DEGs related to signaling and hormone metabolism were analyzed using citrus *actin* and *thioredoxin-like protein YLS8* as reference genes (Tang and Vashisth, 2020).

### 2.5 Phytohormone quantification

Phytohormone concentration (ng g^−1^) was determined from all stages of leaf development (T_1_, T_2_, T_3_, T_4_, and T_5_). After grinding, samples were sent to the Nebraska Center for Biotechnology at the University of Nebraska-Lincoln for analysis of hormones such as auxin (IAA, IAA-Ala, IAA-Asp, Methyl-IAA, and IAA-Trp), cytokinin (cZ, tZ, cZR, and tZR), gibberellins (GA_1_, GA_3_, GA_4_, GA_8_, GA_9_, GA_12_, GA_19_, GA_20_, GA_24_, GA_29_, and GA_53_), abscisic acid (ABA), salicylic acid (SA), jasmonic acid (OPDA, JA, and JA-Ile), and strigolactones (orobanchol, strigol, and 5-deoxystrigol) using liquid chromatography–mass spectrometry-targeted assay (Hung et al., 2016).

## 3 Statistical analysis

The data were analyzed using R-studio (R-version 3.4; R-core team, Vienna, Austria). Two-way analysis of variance (ANOVA) with repeated measures [disease condition (D) × leaf stages (T)] was performed to evaluate the effects of disease condition, development stage, and its interactions on buds, leaf emergence, expansion, and maturation. The mean separation was compared using Tukey’s honestly significant difference (HSD) *post-hoc* test to indicate significant differences. A t-test was performed for differences in tree growth attributes between HLY and HLB-affected trees.

## 4 Results

### 4.1 Physiological attributes

**Figure 2:**
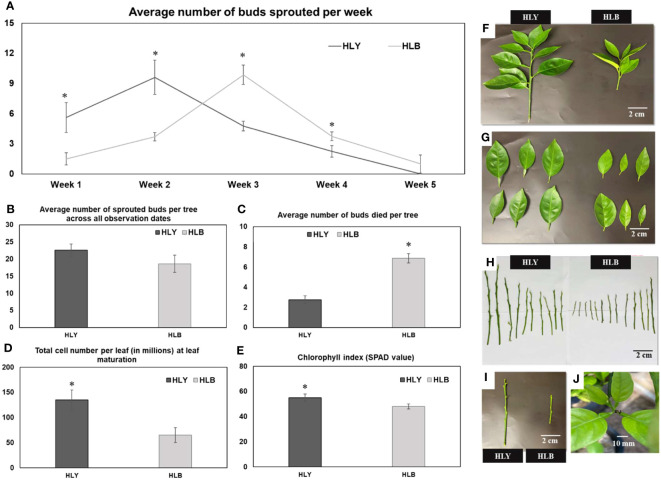
Average number of sprouted buds per week **(A)** Average number of sprouted buds per tree across all observation dates **(B)** Average number of buds died per tree **(C)** Total cell number per leaf (in millions) at leaf maturation stage **(D)** chlorophyll index **(E)** visual shoot growth **(F)** leaf area **(G)** shoot length **(H)** and internode distance **(I)** between healthy (HLY) and Huanglongbing (HLB)-affected sweet orange trees. **(J)** Tips of growing leaves were dead in HLB-affected sweet orange trees. Significant differences were calculated between HLY and HLB-affected sweet orange trees based on *p* < 0.05.

We first compared the physiological attributes of HLB-affected and HLY 7-year-old sweet orange ‘Valencia’ grafted on Swingle citrumelo rootstock in the greenhouse. HLB-affected trees had reduced tree height (48%), number of shoots per tree (40%), shoot length (42%), internode length (35%), leaf size (45%), leaf weight (53%), and SPAD value (25%) compared to HLY trees (**Figures 2A–I**; **Table 1**). The average leaf number per shoot was also lower (five leaves) in HLB-affected trees than in HLY trees (seven leaves) (**Table 1**). HLB-affected trees lagged in bud sprouting as well as growth (approximately 1 week) compared to HLY trees (**Figure 2A**). Bud sprouting peaked at week 2 and week 3 for HLY and HLB-affected trees, respectively. No differences were found in trunk diameter and the total bud emergence per tree at the end of the experiment (**Figure 2B**; **Table 1**). Bud dieback and leaf drop rates were greater in HLB-affected trees (≈10%) compared to HLY trees (<1%), altogether resulting in thinner canopies in HLB-affected trees (**Figures 2C, J**).

**Table 1:** Tree physiological attributes for healthy and HLB-affected trees.

Parameters	Healthy (HLY)	HLB-affected (HLB)	*p*-Value
Physiological attributes			
Individual leaf area (cm^2^)	29.68 a	16.06 b	<0.01
Individual leaf fresh weight (g)	0.76 a	0.36 b	<0.01
Internode length (cm)	1.61 a	1.04 b	<0.01
Shoot length (cm)	13.65 a	7.88 b	<0.01
Number of shoots per tree	9.80 a	6.75 b	<0.01
Number of leaves/shoots	7.5 a	5.75 b	0.01
Increase in tree height (%)	21 a	11 b	0.05
Increase in trunk diameter (%)	2	1	NS*

NS, non-significant; HLB, Huanglongbing. Different letters indicate significant differences among healthy and HLB-affected trees.

### 4.2 Anatomical analysis

**Figure 3:**
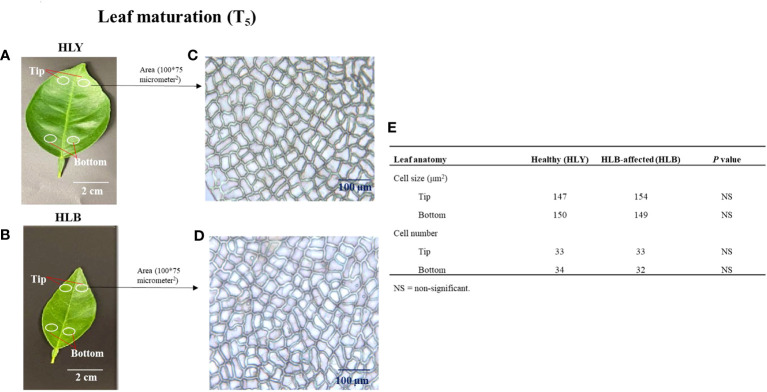
Selection of leaf (3-month-old) areas at tip and bottom sections of healthy (HLY) **(A)** and Huanglongbing (HLB)-affected **(B)** sweet orange trees. Comparative anatomical changes in cell size and cell number within a specified area (100 * 75 μm^2^) from leaves of HLY **(C)** and HLB-affected sweet orange trees **(D)**. Average mean values of cell size and cell number for tip and bottom parts of leaves from HLY and HLB-affected sweet orange trees **(E)**.

Next, we measured the cell sizes and numbers in HLY and HLB-affected trees. No differences were found in leaf cell size and cell number within a specified leaf area (100 * 75 μm^2^) between HLY and HLB-affected trees (**Figures 3A–E**). However, total number of cells per leaf was lower (approximately 50%) in HLB-affected trees compared to HLY trees.

### 4.3 Transcriptome analysis

#### 4.3.1 Differentially expressed genes

A total of 410 and 201 genes were differentially expressed in leaf stages T_2_ (buds collected at day 5) and T_3_ (leaf emergence), respectively, between HLB-affected and HLY trees (**Supplementary Tables S2**, **S3**, **S4**). At stage T_2_, 223 DEGs and 187 DEGs were upregulated and downregulated in HLB-affected trees compared to HLY trees, respectively. At stage T_3_, 188 DEGs were upregulated and 13 DEGs were found downregulated in HLB-affected trees when compared to HLY trees. A significant and positive correlation (R = 0.8) between relative gene expression levels obtained by qPCR and fold changes attained by RNA-seq analysis validated the RNA-seq data (**Supplementary Figure S1**).

#### 4.3.2 Enrichment analysis of DEGs

##### 4.3.2.1 Stage T_2_ (buds at day 5)

**Figure 4:**
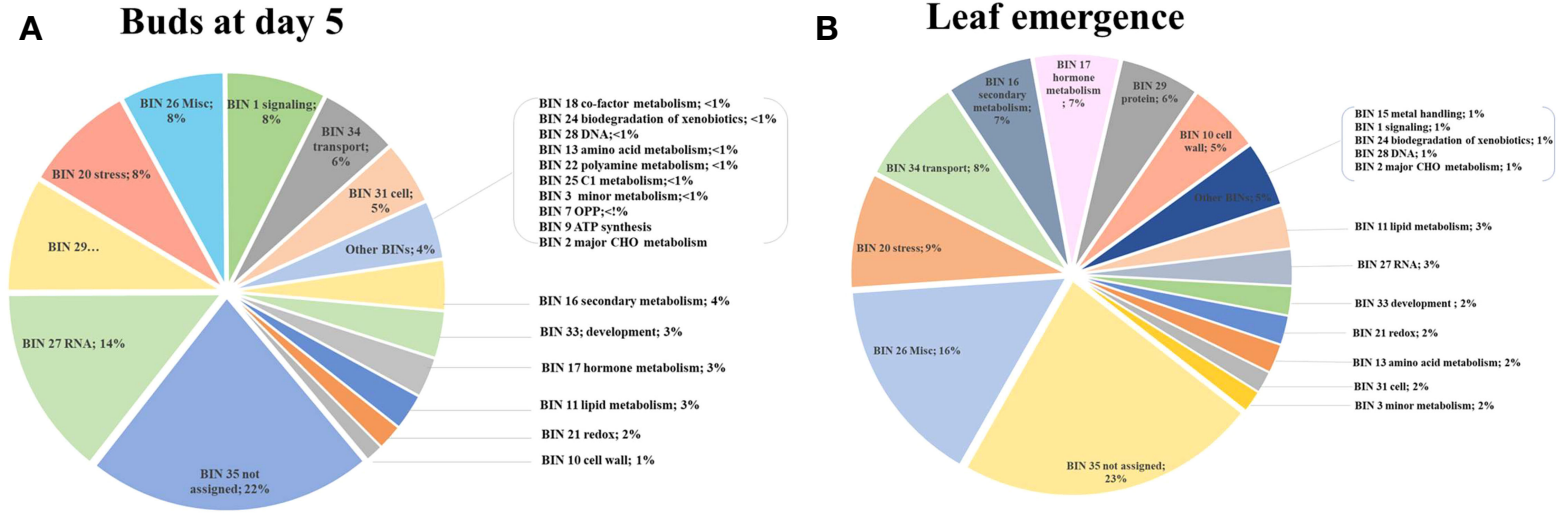
Using MapMan enrichment analysis, major functional BINs via distribution of differentially expressed genes (DEGs) in the buds (T_2_) of Huanglongbing (HLB)-affected trees and healthy sweet orange trees **(A)** and in the newly emerged leaves (T_3_) of Huanglongbing (HLB)-affected trees and healthy sweet orange trees **(B)**.

**Figure 5:**
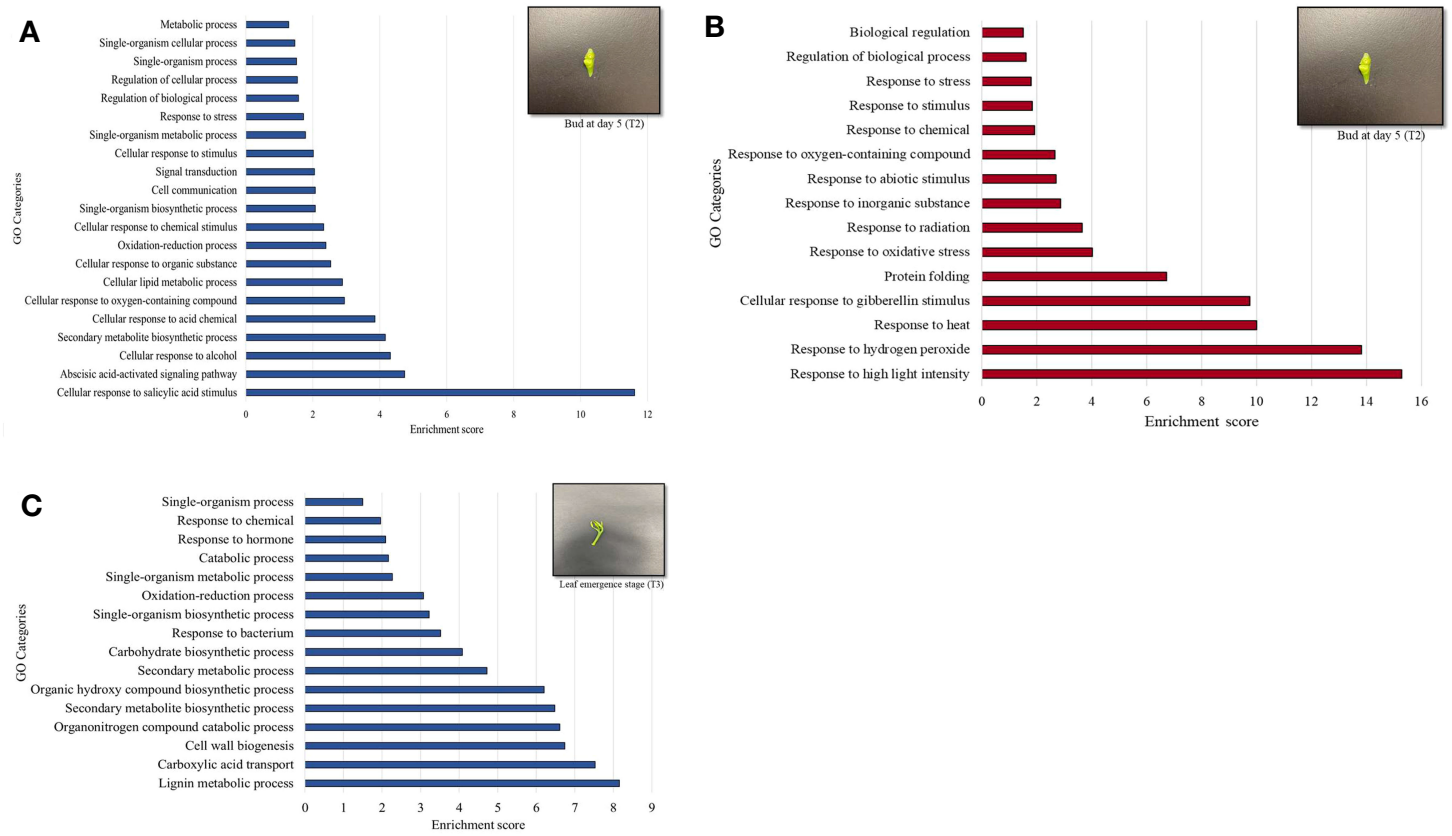
Gene ontology (GO) terms of differentially upregulated genes (DEGs) based on enrichment scores in the buds (T_2_) of Huanglongbing (HLB)-affected trees versus healthy sweet orange trees **(A)**. Downregulated genes (DEGs) based on enrichment scored in the buds (T_2_) of HLB-affected trees versus healthy sweet orange trees **(B)**. Upregulated genes in the newly emerged leaves (T_3_) of HLB-affected trees versus healthy trees **(C)**.

MapMan enrichment analysis revealed an overview of DEGs involved in physiological and metabolic processes in buds of HLY and HLB-affected trees (**Figure 4A**). GO enrichment analysis was further performed using the upregulated and downregulated DEGs separately to reveal the biological process in HLB-affected and HLY trees. For upregulated DEGs in buds of HLB-affected trees in comparison to HLY trees (**Figure 5A**), the top enriched GO terms were related to salicylic acid response (GO:0009751), ABA-activated signaling pathway (GO:0009738), response to alcohol (GO:0097306), secondary metabolite biosynthetic process (GO:0044550), cellular lipid metabolic process (GO:0044255), cellular response to oxygen-containing compound (GO:1901701), oxidation-reduction process (GO:0055114), cell communication (GO:0007154), signal transduction (GO:0007165), and response to stress (GO:0006950). For downregulated DEGs in buds of HLB-affected trees in comparison to HLY trees (**Figure 5B**), the top enriched GO terms were related to high light intensity (GO:0009645), response to hydrogen peroxide (GO:0042542), response to heat (GO:0009408), cellular response to gibberellin stimulus (GO:0071370), protein folding (GO:0006457), response to oxidative stress (GO:0006979), and response to chemical (GO:0042221) (**Figure 5B**). The GO terms response to stress (GO:0006950), response to oxygen-containing compounds (GO:1901700), regulation of biological process (GO:0050789), and response to stimulus (GO:0048583) were found in both the upregulated and downregulated DEGs.

##### 4.3.2.2 Stage T_3_ (leaf emergence)

In stage T_3_ of HLY and HLB-affected trees, DEGs involved in metabolic and physiological processes using MapMan enrichment analysis are shown in **Figure 4B**. Considering GO enrichment analysis for upregulated DEGs in HLB-affected trees compared to HLY trees (**Figure 5C**), the top enriched GO terms were related to lignin metabolic process (GO:0009808), carboxylic acid transport (GO:0046942), cell wall biogenesis (GO:0042546), organonitrogen compound catabolic process (GO:1901565), secondary metabolite biosynthetic process (GO:0044550), oxidation-reduction process (GO:0055114), carbohydrate biosynthetic process (GO:0016051), response to bacterium (GO:0009617), and response to hormone (GO:0009725) (**Figure 5C**).

#### 4.3.3 DEGs related to hormone metabolism and signaling

Out of the mapped DEGs, approximately 3% and 7% DEGs were associated with hormone synthesis in T_2_ (buds at day 5) and T_3_ (newly emerged) leaf stages, respectively (**Figures 4A, B**). In stage T_2_, eight DEGs encoding ABA metabolism, three DEGs encoding ethylene-related proteins, and three DEGs encoding salicylic acid were upregulated in HLB-affected trees compared to HLY trees (**Table 2**). Regarding growth-promoting hormones in HLB-affected trees compared to HLY trees, two DEGs encoding auxin and one DEG encoding cytokinin were upregulated, whereas six DEGs encoding gibberellins were downregulated (**Table 2**).

**Table 2:** Differentially expressed genes (DEGs) related to hormone metabolism and signaling in stage T_2_ (buds at day 5) and T_3_ (leaf emergence) of HLB-affected trees compared to healthy trees.

Process	Stage T_2_ (buds at day 5)				Stage T_3_ (leaf emergence)			
	Citrus ID	*Arabidopsis thaliana* ID	Log_2_ fold	Description	Citrus ID	*Arabidopsis thaliana* ID	Log_2_ fold	Description
Abscisic acid								
	orange1. 1g019546m	AT2G40340	1.28	Dehydration-responsive element binding protein-2 (DREB2C)	orange1. 1g007291m	AT1G30100	1.63	9-*cis*-Epoxy carotenoid dioxygenase 5 (NCED 5)
	orange1. 1g032043m	AT4G33950	1.13	Open stomata 1/SNRK2-6 (OST1)	orange1. 1g029508m	AT1G04120	1.18	Multidrug resistance-associated protein 5 (MRP5)
	orange1. 1g026469m	AT5G47670	2.40	Nuclear factor Y, subunit B6 (NF-YB6)	orange1. 1g032043m	AT2G03440	1.18	Nodulin-related protein 1 (NRP1)
	orange1. 1g029508m	AT1G04120	1.18	Multidrug resistance-associated protein 5 (MRP5)	–	–	–	–
	orange1. 1g015832m	AT2G30020	1.73	Protein phosphatase 2C	–	–	–	–
	orange1. 1g032043m	AT2G03440	1.13	Nodulin-related protein 1 (NRP1)	–	–	–	–
	orange1. 1g028094m	AT5G51990	2.11	C-repeat-binding factor 4 (CBF4)/DREB1	–	–	–	–
	orange1. 1g029527m	AT1G49720	1.47	Abscisic acid-responsive element-binding factor 1 (ABF1)	–	–	–	–
Salicylic acid								
	orange1. 1g044312m	AT1G79680	2.93	Wall-associated kinase-like 10 (WAKL10)	orange1. 1g044312m	AT1G79680	1.56	Wall-associated kinase-like 10 (WAKL10)
	orange1. 1g020291m	AT3G56400	2.24	WRKY DNA-binding protein 70 (WRKY70)	orange1. 1g045300m	AT2G37040	1.98	Phenylalanine ammonia lysase-1 (PAL1)
	orange1. 1g023970m	AT3G01420	1.92	Alpha-dioxygenase (DOX1)	orange1. 1g045376m	AT3G06350	1.69	Maternal effect embryo arrest 32 (MEE32)
Gibberellins								
	orange1. 1g035679m	AT1G75750	−1.93	GAST1 protein homolog 1 (GASA1)	orange1. 1g009215m	AT1G66350	3.71	Repressor of GA (RGA1)
	orange1. 1g042221m	AT4G35390	−2.10	AT-hook protein of GA feedback 1 (AGF1)	orange1. 1g033495m	AT1G74670	1.42	Gibberellin-regulated family protein (GASA 6)
	orange1. 1g041881m	AT1G67030	−1.61	Zinc finger protein 6 (ZFP6)	–	–	–	–
	orange1. 1g005279m	AT1G12240	−2.02	ATBETAFRUCT4	–	–	–	–
Process	Citrus ID	*Arabidopsis thaliana* ID	Log_2_ fold	Description	Citrus ID	*Arabidopsis thaliana* ID	Log_2_ fold	Description
	orange1. 1g004262m	AT1G01060	−1.81	Late elongated hypocotyl (LHY)	–	–	–	–
	orange1. 1g033766m	AT2G03500	−1.34	Early flowering MYB protein	–	–	–	–
Cytokinins								
	orange1. 1g048204m	AT1G03430	1.08	Histidine-containing phosphotransferase factor 5 (AHP5)	orange1. 1g048204m	AT1G03430	1.10	Cytokinins. Histidine-containing phosphotransferase factor 5 (AHP5)
Ethylene								
	orange1. 1g004510m	AT3G04580	1.56	Ethylene insensitive 4 (EIN4)	orange1. 1g025382m	AT3G16770	1.56	Ethylene-responsive element binding factor (ERF38)
	orange1. 1g029068m	AT1G19210	2.48	Ethylene-responsive element binding factor (ERF17)	orange1. 1g044843m	AT4G01850	1.33	*S*-Adenosylmethionine synthetase 2 (SAM 2)
	orange1. 1g030002m	AT5G07580	2.62	Ethylene-responsive element binding factor (ERF106)	orange1. 1g011801m	AT3G61510	3.80	ACC synthase 1
Auxins								
	orange1. 1g013651m	AT4G31500	2.10	Cytochrome P450, family 83, subfamily B, polypeptide 1 (CYP83B1)	orange1. 1g013651m	AT4G31500	1.89	Cytochrome P450, family 83, subfamily B, polypeptide 1 (CYP83B1)
	orange1. 1g042071m	AT3G08510	1.91	Phosphoinositide-specific phospholipase C 2 (PLC2)	orange1.1g046614m	AT1G29450	3.88	SAUR-like auxin-responsive protein family

Similar to T_2_, at leaf stage T_3_, three DEGs encoding ABA metabolism, three DEGs encoding salicylic acid biosynthesis/degradation, and three DEGs encoding ethylene-related proteins were upregulated in HLB-affected trees compared to HLY trees (**Table 2**). Two DEGs encoding auxin, one DEG encoding cytokinin, and two DEGs encoding gibberellins were upregulated in HLB-affected trees compared to HLY trees (**Table 2**).

#### 4.3.4 DEGs involved in leaf development processes

At stage T_2_ (buds), DEG related to *DA1-related protein 2* was downregulated in HLB-affected trees compared to HLY trees (**Table 3**). Three DEGs related to *growth-regulating factor* (GRF-GIF) were upregulated in HLB-affected trees compared to HLY trees (**Table 3**).

**Table 3:** Differentially expressed genes (DEGs) related to leaf development in stage T_2_ (buds at day 5) and T_3_ (leaf emergence) of Huanglongbing (HLB)-affected trees compared to healthy trees.

Module		Stage T_2_ (buds at day 5)				Stage T_3_ (leaf emergence)			
		Citrus ID	*Arabidopsis thaliana* ID	Log_2_ fold	Description	Citrus ID	*Arabidopsis thaliana* ID	Log_2_ fold	Description
Cell expansion		– – – –	– – – –	– – – –		orange1.1g009414m orange1.1g025740m orange1.1g025347m orange1.1g025298m	AT2G46660 AT1G69530 AT2G40610 AT1G20190	1.52 1.21 2.12 3.38	Cytochrome P450, family 78, subfamily A, polypeptide 6 Expansin A1 Expansin A8 Expansin 11
DA1–EOD1		orange1.1g010581m	AT2G39830	−1.26	DA1-related protein 2	–	–	–	
GA–DELLA		–	–	–		orange1.1g009215m	AT1G66350	3.72	RGA-like 1
GRF–GIF		orange1.1g044451m orange1.1g036176m orange1.1g047108m	AT2G36400 AT2G36400 AT3G13960	1.09 1.11 1.65	Growth-regulating factor 3 Growth-regulating factor 3 Growth-regulating factor 5	– – –	– – –	– – –	
KLU		–	–	–		orange1.1g047310m	AT1G13710	1.26	Cytochrome P450, family 78, subfamily A, polypeptide 5

At stage T_3_ (leaf emergence), four DEGs related to *expansin* were upregulated in HLB-affected trees compared to HLY trees (**Table 3**). One DEG related to *RGA-like 1* (GA–DELLA) and another DEG related to *KLU* (cytochrome P450) were upregulated in HLB-affected trees compared to HLY trees (**Table 3**).

#### 4.3.5 DEGs related to oxidative stress and cell death

At stage T_2_, in regard to the *redox state*, five DEGs were upregulated and two DEGs were downregulated in HLB-affected trees compared to HLY trees (**Table 4**). Regarding *peroxidases*, two DEGs were upregulated and one DEG was downregulated in HLB-affected trees compared to HLY trees (**Table 4**). One DEG related to *glutathione S-transferase* was downregulated in HLB-affected trees compared to HLY trees (**Table 4**). Three DEGs related to *senescence* were upregulated in HLB-affected trees compared to HLY trees (**Table 4**).

**Table 4:** Differentially expressed genes (DEGs) related to redox state, peroxidases, glutathione *S*-transferase, cell death, PP2, and transcription factor (WRKY) in stage T_2_ (buds at day 5) and T_3_ (leaf emergence) of Huanglongbing (HLB)-affected trees compared to healthy trees.

	Stage T_2_ (buds at day 5)				Stage T_3_ (leaf emergence)			
Process	Citrus ID	*Arabidopsis thaliana* ID	Log_2_ fold	Description	Citrus ID	*Arabidopsis thaliana* ID	Log_2_ fold	Description
**Redox state**	orange1.1g031271m orange1.1g029703m orange1.1g023089m orange1.1g016453m orange1.1g039608m orange1.1g034075m	AT1G59730 AT5G06690 AT5G61440 AT5G20140 AT1G03020 AT5G18600	−1.73 3.41 1.75 −1.01 −2.79 −2.88	Thioredoxin H-type 7 WCRKC thioredoxin 1 Atypical CYS-HIS rich thioredoxin 5 SOUL heme-binding family protein Thioredoxin superfamily protein Thioredoxin superfamily protein	orange1.1g023089m orange1.1g038785m orange1.1g006865m orange1.1g031837m – –	AT5G61440 AT3G19000 AT4G29210 AT1G08830 – –	−1.09 1.52 1.63 −1.03 – –	Atypical CYS HIS rich thioredoxin 5 2-Oxoglutarate (2OG) and Fe(II)-dependent oxygenase superfamily protein Gamma-glutamyl transpeptidase 4 Copper/zinc superoxide dismutase 1
**Peroxidases**	orange1.1g020451m orange1.1g019278m orange1.1g018847m	AT1G49570 AT1G71695 AT5G06720	2.31 1.12 −2.15	Peroxidase superfamily protein Peroxidase superfamily protein Peroxidase 2	orange1.1g009835m orange1.1g037904m orange1.1g048141m	AT2G34930 AT5G45200 AT1G17860	1.75 −1.47 4.39	Disease resistance family protein/LRR family protein Disease resistance protein (TIR-NBS-LRR class) family Kunitz family trypsin and protease inhibitor protein
**Glutathione *S*-transferase**	orange1.1g027827m	AT3G09270	−1.11	Glutathione *S*-transferase TAU 8	–	–	–	
**Cell death**	orange1.1g035879 morange1.1g045644m orange1.1g036388m – – –	AT1G17020 AT1G17020 AT2G29350 – – –	1.39 1.40 1.29 – – –	Senescence-related gene 1 Senescence-related gene 1 Senescence-associated gene 13	orange1. 1g045644m orange1. 1g036388m orange1. 1g013951m orange1. 1g031484m orange1. 1g032569m orange1. 1g040925m	AT4G33980 AT3G24420 AT5G65300 AT5G19650 AT4G39400 AT5G43650	1.39 1.28 2.0 1.29 2.36 3.74	Senescence-related gene 1 Senescence-associated gene 13 UDP-Glycosyltransferase superfamily protein UDP-Glycosyltransferase superfamily protein Leucine-rich receptor-like protein kinase family protein Leucine-rich repeat protein kinase family protein
**PP_2_**	orange1.1g042543m orange1.1g045590m	AT1G56240 AT1G09155	5.90 7.40	Phloem protein 2-B13 Phloem protein 2-B15	orange1.1g042543m orange1.1g045590m	AT1G56240 AT1G09155	5.68 4.33	Phloem protein 2-B13 Phloem protein 2-B15
**WRKY**	orange1.1g020291m orange1.1g032690m orange1.1g029257m	AT3G56400 AT2G47260 AT5G64810	2.25 1.20 3.54	WRKY DNA-binding protein 70 WRKY DNA-binding protein 23 WRKY DNA-binding protein 51	– – –	– – –	– – –	

At stage T_3_, out of four, two DEGs related to the redox state were upregulated, whereas the other two DEGs were downregulated in HLB-affected trees compared to HLY trees (**Table 4**). With respect to *peroxidases*, two DEGs were upregulated and one DEG was downregulated in HLB-affected trees compared to HLY trees (**Table 4**). All six DEGs related to *senescence* and *cell death* were upregulated in HLB-affected trees in comparison to HLY trees (**Table 4**).

### 4.4 Phytohormone analysis

**Figure 6:**
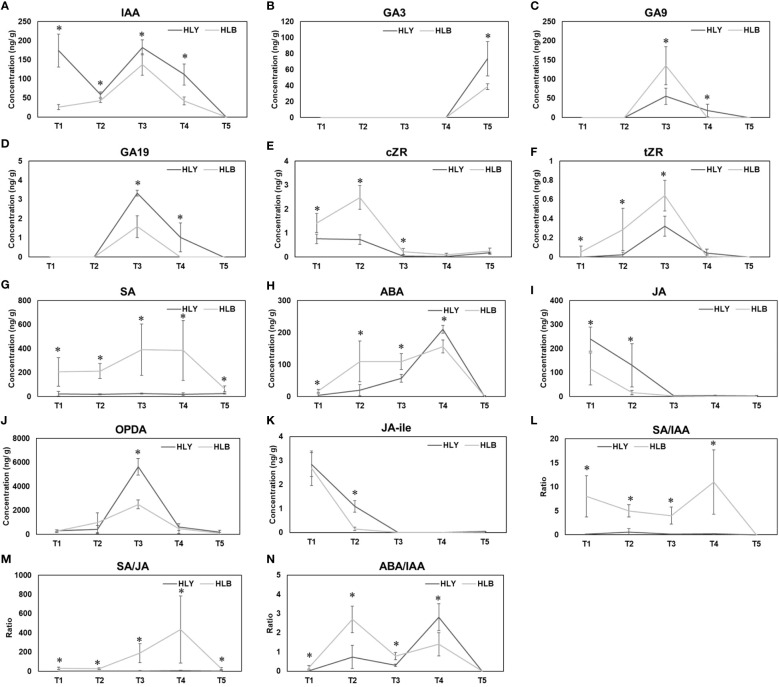
Phytohormone concentrations and hormonal ratios from bud emergence to leaf maturation between healthy and Huanglongbing **(HLB)**-affected sweet orange trees. T_1_, buds at the start of experiment; T_2_, buds on day 5; T_3_, leaf emergence; T_4_, leaf expansion; T_5_, leaf maturation. IAA **(A)** GA3 **(B)** GA9 **(C)** GA19 **(D)** cZR **(E)** tZR **(F)** SA **(G)** ABA **(H)** JA **(I)** OPDA **(J)** JA-ile **(K)** SA/IAA **(L)** SA/JA **(M)** ABA/IAA **(N)**. Significant differences were calculated between HLY and HLB-affected sweet orange trees based on *p* < 0.05.

For growth-promoting hormones, HLB-affected trees had lower leaf IAA content compared to HLY trees. Leaves from HLY trees had more IAA than HLB-affected trees in stage T_1_ (6.6-fold), T_2_ (1.4-fold), T_3_ (1.3-fold), and T_4_ (2.7-fold) (**Figure 6A**). IAA was not detected at T_5_ (leaf maturation) in both HLY and HLB-affected trees. Considering gibberellins, GA_3_ (active form) was only detected in stage T_5_ (leaf maturation) and was 1.9-fold lower in HLB-affected trees than in HLY trees (**Figure 6B**). For GA (inactive form), HLB-affected trees had higher GA_9_ (2.4-fold) and lower GA_19_ (2.1-fold) compared to HLY trees, and maximum concentrations were detected in stage T_3_ (leaf emergence) (**Figures 6C, D**). GA was not detected in stages T_1_ and T_2_ (buds) for both HLY and HLB-affected trees. Cytokinin (cZR) was found to be high in stages T_1_ (1.9-fold), T_2_ (3.4-fold), and T_3_ (5.4-fold) for HLB-affected trees compared to HLY trees, and a similar trend was observed for tZR content (**Figures 6E, F**).

For defense-related hormones, SA was found higher in HLB-affected trees in stages T_1_ (9.6-fold), T_2_ (11.7-fold), T_3_ (15.7-fold), T_4_ (20.2-fold), and T_5_ (2.6-fold) compared to that in HLY trees (**Figure 6G**). HLB-affected trees had higher ABA in stages T_1_ (4.6-fold), T_2_ (5.6-fold), and T_3_ (19.1-fold) than HLY trees (**Figure 6H**). On T_4_, HLY trees had higher ABA (1.8-fold) compared to HLB-affected trees, and no ABA content was found in stage T_5_ of both HLY and HLB-affected trees. HLB-affected trees had lower JA in stages T_1_ (2.1-fold) and T_2_ (8.1-fold) in comparison to HLY trees (**Figure 6I**). HLB-affected trees also had lower OPDA (JA precursor) on T_3_ (2.3-fold) compared to HLY trees (**Figure 6J**). Strigolactones were not detected during leaf development at any time point. Overall, HLB-affected trees had higher SA, ABA, and cytokinin and lower IAA, GA_3_, and JA content compared to HLY trees.

Considering hormonal ratios, HLB-affected trees had higher SA/IAA ratios in stages T_1_ (75.2-fold), T_2_ (8.4-fold), T_3_ (22.5-fold), and T_4_ (52.9-fold) compared to HLY trees (**Figure 6L**). SA/JA ratios were higher in HLB-affected trees in stages T_1_ (70.7-fold), T_2_ (19.6-fold), T_3_ (55.9-fold), T_4_ (65.3-fold), and T_5_ (5.5-fold) than in HLY trees (**Figure 6M**). ABA/IAA ratios were higher in HLB-affected trees in stages T_1_ (52.1-fold), T_2_ (3.7-fold), T_3_ (2.5-fold), and T_4_ (2.0-fold) compared to HLY trees (**Figure 6N**).

## 5 Discussion

Herein, we present a comparative study between HLY and HLB-affected trees exploring the morphological characteristics, followed by transcriptome and hormone analyses, to understand the mechanism behind poor growth traits in HLB-affected trees. HLB incidence resulted in ≈40% reduction in growth including tree height, number of shoots per tree, shoot length, shoot internode distance, fewer number of leaves per shoot, and leaf size when compared to HLY trees. Furthermore, a delay in bud sprouting (≈1 week) and increased bud dieback and leaf drop were observed in HLB-affected trees than in HLY trees. This is the first study that presents leaf and shoot level morphological characteristics in HLB-affected trees and is further supported by transcriptome and phytohormone levels; thus, it is quite likely that poor leaf and shoot growth lead to overall poor canopy (sparse) and a decline in fruit production.

Generally, phytohormones can be categorized as growth-promoting (auxin, GA, and cytokinin) and defense-related (SA, ABA, JA, and ethylene) hormones, and often, an antagonistic relationship exists between growth and defense-related hormones. Upon *C*Las infection, an increase in leaf SA has been reported in sweet orange (Nehela et al., 2018; Shahzad et al., 2023). Similarly, our results showed 10 times higher SA in buds of HLB-affected trees compared to HLY trees, which increased up to 20% as the leaves grew, along with upregulated DEGs encoding SA biosynthesis. Salicylic acid activates systemic acquired resistance response against *C*Las bacteria as they create the site for new infection (Ma et al., 2022) by increasing the expression of WRKY transcription factors, PR proteins, and secondary metabolites (Mafra et al., 2013; Hu et al., 2017); these observations are consistent with our results. SA also represses auxin signaling, as some plant pathogens produce auxin to manipulate the host development processes (Manulis et al., 1998), and our results also showed higher SA/IAA ratios in leaf stages T_2_ (8.4-fold) and T_3_ (22.5-fold) of HLB-affected trees compared to HLY trees. Auxins are essential for all stages of leaf development, and a lower auxin level was found in buds and growing leaves of HLB-affected trees than in HLY trees. Contrary to our findings, higher levels of auxin have been reported in HLB-affected sweet orange leaves by Nehela et al. (2016). However, as the main goal of their study was to evaluate the hormone profile at the tree level, juvenile leaves were pooled together with older leaves on the collection day. However, in the present study, auxin was quantified at each phenological leaf stage. In contrast, higher levels of ABA in leaf stages in T_1_ (4.6-fold), T_2_ (5.6-fold), and T_3_ (19.1-fold) as well as DEGs encoding ABA biosynthesis/degradation were found upregulated in HLB-affected trees in comparison to HLY trees. Furthermore, higher ABA/IAA ratios were found in stages T_1_ (52.1-fold), T_2_ (3.7-fold), and T_3_ (2.5-fold) of HLB-affected trees than HLY trees. HLB-affected trees are known to undergo root dieback, thereby possibly limiting the water uptake (Hamido et al., 2019). An increase in water deficit positively correlates with HLB severity (Tang and Vashisth, 2020); therefore, high ABA accumulation as a response to water deficit is expected in HLB-affected trees. Plant responses to drought including stomatal closure are regulated by ABA, which plays a key role in modulating the intensity of the physiological response to the stress (Gómez-Cadenas et al., 1996). Endogenous ABA accumulation had a growth inhibitory effect that resulted in arrested leaf growth as documented in different crops (Milborrow, 1974). In this study, higher ABA concentration in stages T_1_, T_2_, and T_3_ of HLB-affected trees would explain the delay in bud sprouting as well as the small leaf size. de Ollas et al. (2013) have shown that ABA and JA interact at the biosynthetic level in citrus where transient JA accumulation is necessary for subsequent ABA increase when the tree is under drought stress. Similar to their finding, in this study, a short-lived burst in OPDA accumulation (JA precursor) was seen in T_3_ of HLY trees (**Figures 6I–K**), which may have boosted the ABA accumulation during leaf development (on T_4_). This observation of higher ABA in T_4_ of HLY trees may coincide with the competition of leaf development and initiate signaling for arrested leaf growth via stress-induced stomatal closure. Growth-promoting hormones (auxins, GA, and cytokinin) contribute to leaf growth through cell proliferation and expansion (Ueguchi-Tanaka et al., 2007; Zhao et al., 2021). Auxin regulates all stages of leaf development (Zhao et al., 2021), and high auxin often coincides with leaf primordia initiation (Dong and Huang, 2018). Regarding GA, genes related to GA biosynthesis and signaling like GAST1 protein homolog 1 (GASA1) and AT-hook protein of GA feedback 1 (AGF1) were downregulated, and repressor of GA (RGA1) and gibberellin-regulated family protein (GASA 6), which negatively affects the GA biosynthesis, were upregulated in HLB-affected trees. Concomitantly, a low GA_3_ level with reduced leaf size and shoot growth was found in HLB-affected trees compared to HLY trees. In HLB-affected trees, auxin positively modulates GA synthesis, which activates the response mechanism to *C*Las infection such as callose, PP_2_ deposition, and impaired substance transport leading to HLB symptom development, while in the tolerant variety, suppression of the auxin pathway prevents the events leading to phloem dysfunction (Curtolo et al., 2020). Leaf development is a dynamic and multifactorial process involving many genes that regulate final leaf size. DEGs encoding cell expansion were upregulated in HLB-affected trees compared to HLY trees. Expansin proteins loosen the cell wall structure by cell wall extension in water-deficit conditions, resulting in the stiffening of leaves and distortion of the phloem cell wall (Folimonova and Achor, 2010; Musetti et al., 2010), which are characteristic HLB symptoms. In the DA1-EOD1 module, enhancer DA1-EOD1 limits the duration for cell proliferation (Li et al., 2008), and DEG encoding DA1-EOD1 was downregulated in HLB-affected trees compared to HLY trees, which are indicators for improved cell expansion. In the GRF-GIF module, GRFs are enriched in meristematic tissues and positive regulators for cell proliferation (Kim and Kende, 2004), and DEG encoding growth-regulating factor 3 and growth-regulating factor 5 were upregulated in HLB-affected trees compared to HLY trees. The KLU, a plant-specific cytochrome P450 protein belonging to the CYP78A subfamily, regulates leaf size by cell proliferation (Eriksson et al., 2010). DEG encoding KLU was found upregulated in HLB-affected trees compared to HLY trees. Altogether, HLB-affected trees had upregulated DEGs that are involved in leaf development by accentuating cell proliferation and expansion as well as low levels of auxin and GA as compared to HLY trees. Thus, it can be speculated that low levels of growth-promoting hormone resulted in overall compromised leaf and shoot growth. However, no differences in leaf anatomy at the cell level (cell number and cell size) were observed, as the cell proliferation and expansion were not affected by HLB. Taken together, lower auxin and GA (growth-promoting) levels and higher SA and ABA levels (defense) explain the delay in the bud sprouting and poor leaf and shoot growth attributes as the HLB-affected trees tradeoff resources for defense response to *C*Las and water deficit (a symptom of HLB). Recently, the use of exogenous GA_3_ has been shown to be beneficial for HLB-affected trees in promoting vegetative growth and fruit yield (Singh et al., 2022), suggesting that supplementing growth-promoting hormones can help in overcoming HLB-induced hormonal imbalance.

The senescence process in plants is categorized as 1) mitotic (occurs in shoot apical meristem containing multipotent stem cells) and 2) postmitotic (occurs in organs such as leaves and flowers) (Guo and Gan, 2005). Leaf senescence begins when the photosynthetic rate drops below a certain threshold level, nutrient uptake decreases, and chlorophyll, lipid, DNA, and RNA degrades. Finally, the leaves do not contribute to carbon fixation. Higher levels of defense (SA, ABA, and ethylene) and lower levels of growth-promoting (auxin and GA) hormones might trigger both mitotic (bud dieback) and postmitotic (accelerated leaf aging and leaf drop) senescence in HLB-affected trees than HLY trees. In *Arabidopsis*, SA mediates stress response by enhancing reactive oxygen species (ROS) production, which results in a gradual increase in leaf senescence by inducing autophagic lysosome formation (Zhang et al., 2017; Yin et al., 2020). In water-deficit conditions, higher ABA also triggers leaf senescence by promoting ethylene production as documented in Cleopatra mandarin (Gómez-Cadenas et al., 1996; Iqbal et al., 2017; Shahzad et al., 2023). In our study, DEGs encoding ethylene-related proteins were upregulated in HLB-affected trees compared to HLY trees. Ma et al. (2022) reported that HLB is a *C*Las-triggered immune response disease that is associated with excessive ROS production and accumulation. Our results suggested that HLB is not only linked to immunity itself, but HLB severity comes at the expense of growth (decrease in auxin and GA) via high investment in defense (increase in SA and ABA) against *C*Las. It is possible that defense hormones (SA and ABA) trigger ROS accumulation (Ton et al., 2009) and ROS intermediates redox signaling and oxidation-reduction in host plant resistance (Shi et al., 2019). DEG encoding glutathione *S*-transferases and DEGs encoding redox state were also downregulated in HLB-affected trees compared to HLY trees, suggesting that HLB-affected trees are prone to oxidative stress. Also, upregulated DEGs encoding senescence and GO term related to cell death in HLB-affected trees compared to HLY trees are indicators for accelerated bud dieback and leaf drop resulting in sparse canopies.

## 6 Conclusions

Our results present evidence that upon *C*Las infection, HLB-affected trees are lagging in bud emergence along with growth by approximately 1 week, which contributes to compromised leaf and shoot development as well as higher bud dieback resulting in thinner canopies and reduced life span with HLB progression. During leaf development phases, higher SA/IAA and ABA/IAA rations were found in leaves of HLB-affected trees compared to HLY trees, suggesting more tradeoff of resources on defense over growth in HLB-affected trees. Leaf anatomy results show that the total cell number per leaf was lower in HLB-affected trees compared to HLY trees, which may coincide with low levels of growth-promoting hormones (auxin and GA_3_). Often, defense and growth-related hormones are antagonistic. Our results suggest that reduction in growth in HLB-affected trees may result from increased and constant investment of the trees in the defense against *C*Las.

## Data availability statement

The datasets presented in this study can be found in online repositories. The names of the repository/repositories and accession number(s) can be found below: NCBI, PRJNA1042812.

## Author contributions

AN: Formal analysis, Investigation, Methodology, Writing – original draft. FS: Formal analysis, Validation, Data Curation, Writing – original draft. CB: Methodology, Writing – review & editing. AL: Methodology, Writing – review & editing. TV: Conceptualization, Funding acquisition, Methodology, Project administration, Supervision, Writing – review & editing.
