# Supplementary material for: Poor shoot and leaf growth in Huanglongbing-affected sweet orange is associated with increased investment in defenses
Source: Front Plant Sci. 2023 Dec 19;14:1305815. doi: 10.3389/fpls.2023.1305815 (PMC10766359; doi:10.3389/fpls.2023.1305815)
Supplement: Supplementary file 2 [file DataSheet_1.docx]

| **Supplemental Table S1**. Gene-specific primer sequences for relative gene expression analysis during leaf development stages T_2_ (buds at day 5) and T_3_ (leaf emergence) of Huanglongbing (HLB)-affected and healthy sweet orange trees, with quantitative real-time polymerase chain reaction (qPCR) for RNA-sequencing data validation. | | | |
| --- | --- | --- | --- |
| **Gene symbol** | **Description** | **GenBank accession no.** | **Forward and reverse primer sequences (5’ to 3’)** |
| *CAM8* | calmodulin 8 | XM_006489415.3 | TGACGCTGAGGAAGAGCTTAAA |
|  |  |  | ATCACCGTCCAAATCAGCCTC |
| *SAG20* | Senescence associated gene 20 | XM_006486163.3 | AAAGCCATCACCAGTTCTC |
|  |  |  | GTTCTTGACGGAATAGCCTTA |
| *ERF1* | Ethylene response factor 1 | XM_006477063.3 | ACGTAGCGCTTCTCAAACCC |
|  |  |  | GCCAATGACCACGATGTTGA |
| *OFP* | Oxidoreductase family protein | XM_006477097.3 | TGGTTCTCGAGGGATGCTTG |
|  |  |  | TCTCAGGAACAAAGGCCTCG |
| *ARF* | Auxin-response factor | NM_001288860.1 | TTCGTGTTGGTGTAAGGCGA |
|  |  |  | TGATGGGCTTGTTCTTGGCT |
| *IAA9* | Indole-2 acetic acid inducible 9 | XM_006466306.3 | ATGGTGACTGGATGCTCGTT |
|  |  |  | TTTTTCCATTGCCCTGGGAG |
| *ARF16* | Auxin response factor 16 | XM_006472629.2 | GGAGTTTCGCCACATTTAT |
|  |  |  | TCCATTGTTTCCGTACCT |
| *ABF3* | Auxin-responsive family protein | XM_006487050.3 | AGCGGTGCTCTGGAGAAAG |
|  |  |  | CTCTGCCTGTTTCCTCTCCAA |
| *ACT7z* | Actin 7 | XM_006464503.2 | GTTGGTTGACATGGAGAAG |
|  |  |  | GACGGTTGAGTACAGAAATAAG |
| *DIM1* | Thioredoxin-like protein YLS8 | XM_006484463.2 | CGAAACCTGTATGCAGATGG |
|  |  |  | ACGGTTGAGGGATCGTAAAG |
| z*ACT* and *DIM1* were used as internal controls for relative gene expression analysis with qPCR. | | | |

| **Supplemental Table S2.** Differentially expressed genes (DEGs) in stage T_2_ (buds at day 5) of Huanglongbing (HLB)-affected trees compared to healthy trees. | | | | |
| --- | --- | --- | --- | --- |
| **DEG No** | **DEG**  **(*Citrus sinensis*)** | ***Arabidopsis thaliana* ortholog** | **log_2_ Fold Change** | **Description** |
| **Upregulated DEGs** | | | | |
| 1 | orange1.1g042686m | AT5G53130 | 8.751699917 | cyclic nucleotide gated channel 1 |
| 2 | orange1.1g045590m | AT1G09155 | 7.401230943 | phloem protein 2-B15 |
| 3 | orange1.1g042543m | AT1G56240 | 5.899906382 | phloem protein 2-B13 |
| 4 | orange1.1g032466m | AT3G26740 | 5.603820392 | CCR-like |
| 5 | orange1.1g039313m | AT5G53360 | 5.543415469 | TRAF-like superfamily protein |
| 6 | orange1.1g036238m | AT3G48030 | 5.204248615 | hypoxia-responsive family protein / zinc finger (C3HC4-type RING finger) family protein |
| 7 | orange1.1g018051m | AT3G12750 | 5.111380124 | zinc transporter 1 precursor |
| 8 | orange1.1g030672m | AT3G62550 | 4.5477133 | Adenine nucleotide alpha hydrolases-like superfamily protein |
| 9 | orange1.1g048792m | AT3G02645 | 4.174079712 | Plant protein of unknown function (DUF247) |
| 10 | orange1.1g028325m | AT2G15890 | 4.074484214 | maternal effect embryo arrest 14 |
| 11 | orange1.1g018188m | AT5G54770 | 3.97348548 | thiazole biosynthetic enzyme, chloroplast (ARA6) (THI1) (THI4) |
| 12 | orange1.1g045669m | AT3G54540 | 3.973289358 | general control non-repressible 4 |
| 13 | orange1.1g032847m | AT3G25882 | 3.911371947 | NIM1-interacting 2 |
| 14 | orange1.1g020458m | AT1G13110 | 3.745641221 | cytochrome P450, family 71 subfamily B, polypeptide 7 |
| 15 | orange1.1g040925m | AT3G47570 | 3.74061243 | Leucine-rich repeat protein kinase family protein |
| 16 | orange1.1g025320m | AT1G07050 | 3.708112621 | CCT motif family protein |
| 17 | orange1.1g029257m | AT5G64810 | 3.541923774 | WRKY DNA-binding protein 51 |
| 18 | orange1.1g010747m | AT4G10770 | 3.464142372 | oligopeptide transporter 7 |
| 19 | orange1.1g030629m | AT4G37300 | 3.46310804 | maternal effect embryo arrest 59 |
| **DEG No** | **DEG**  **(*Citrus sinensis*)** | ***Arabidopsis thaliana* ortholog** | **log_2_ Fold Change** | **Description** |
| 20 | orange1.1g048376m | AT3G48310 | 3.462491164 | cytochrome P450, family 71, subfamily A, polypeptide 22 |
| 21 | orange1.1g029703m | AT5G06690 | 3.412537568 | WCRKC thioredoxin 1 |
| 22 | orange1.1g039456m | AT4G33980 | 3.189922763 | - |
| 23 | orange1.1g037784m | AT1G03220 | 3.153606283 | Eukaryotic aspartyl protease family protein |
| 24 | orange1.1g039815m | AT3G11600 | 3.117185944 | - |
| 25 | orange1.1g010376m | AT5G63850 | 3.081262635 | amino acid permease 4 |
| 26 | orange1.1g017150m | AT4G05200 | 3.067449455 | cysteine-rich RLK (RECEPTOR-like protein kinase) 25 |
| 27 | orange1.1g012938m | AT1G75430 | 3.006213779 | BEL1-like homeodomain 11 |
| 28 | orange1.1g043602m | AT3G18010 | 2.9891246 | WUSCHEL related homeobox 1 |
| 29 | orange1.1g019816m | AT4G03460 | 2.969188685 | Ankyrin repeat family protein |
| 30 | orange1.1g008977m | AT4G03490 | 2.963777532 | Ankyrin repeat family protein |
| 31 | orange1.1g000475m | AT1G04120 | 2.953144011 | multidrug resistance-associated protein 5 |
| 32 | orange1.1g044312m | AT1G79680 | 2.935362089 | WALL ASSOCIATED KINASE (WAK)-LIKE 10 |
| 33 | orange1.1g040769m | AT5G03790 | 2.931630956 | homeobox 51 |
| 34 | orange1.1g046822m | AT1G04150 | 2.854564841 | C2 calcium/lipid-binding plant phosphoribosyltransferase family protein |
| 35 | orange1.1g005920m | AT5G24470 | 2.732276064 | pseudo-response regulator 5 |
| 36 | orange1.1g047842m | AT2G23180 | 2.681121485 | cytochrome P450, family 96, subfamily A, polypeptide 1 |
| 37 | orange1.1g030002m | AT5G07580 | 2.626983792 | Integrase-type DNA-binding superfamily protein |
| 38 | orange1.1g034278m | AT1G52240 | 2.625294417 | RHO guanyl-nucleotide exchange factor 11 |
| 39 | orange1.1g017973m | AT3G51290 | 2.616379458 | Protein of unknown function (DUF630) ;Protein of unknown function (DUF632) |
| 40 | orange1.1g040581m | AT5G19120 | 2.593446518 | Eukaryotic aspartyl protease family protein |
| **DEG No** | **DEG**  **(*Citrus sinensis*)** | ***Arabidopsis thaliana* ortholog** | **log_2_ Fold Change** | **Description** |
| 41 | orange1.1g045379m | AT2G35130 | 2.582791345 | Tetratricopeptide repeat (TPR)-like superfamily protein |
| 42 | orange1.1g024033m | AT3G24420 | 2.524613067 | alpha/beta-Hydrolases superfamily protein |
| 43 | orange1.1g029068m | AT1G19210 | 2.481854119 | Integrase-type DNA-binding superfamily protein |
| 44 | orange1.1g016330m | AT5G13930 | 2.480889802 | Chalcone and stilbene synthase family protein |
| 45 | orange1.1g009495m | AT4G12300 | 2.468712873 | cytochrome P450, family 706, subfamily A, polypeptide 4 |
| 46 | orange1.1g048754m | AT1G68050 | 2.436080266 | flavin-binding, kelch repeat, f box 1 |
| 47 | orange1.1g026469m | AT5G47670 | 2.407731087 | nuclear factor Y, subunit B6 |
| 48 | orange1.1g020537m | AT4G03500 | 2.400928578 | Ankyrin repeat family protein |
| 49 | orange1.1g026746m | AT5G43650 | 2.365321252 | basic helix-loop-helix (bHLH) DNA-binding superfamily protein |
| 50 | orange1.1g032569m | AT5G46330 | 2.364401561 | Leucine-rich receptor-like protein kinase family protein |
| 51 | orange1.1g023278m | AT3G15840 | 2.329556014 | post-illumination chlorophyll fluorescence increase |
| 52 | orange1.1g020451m | AT1G49570 | 2.31069627 | Peroxidase superfamily protein |
| 53 | orange1.1g037671m | AT5G67140 | 2.306156959 | F-box/RNI-like superfamily protein |
| 54 | orange1.1g003267m | AT3G02060 | 2.281111173 | DEAD/DEAH box helicase, putative |
| 55 | orange1.1g013209m | AT2G46210 | 2.263167365 | Fatty acid/sphingolipid desaturase |
| 56 | orange1.1g020291m | AT3G56400 | 2.246367964 | WRKY DNA-binding protein 70 |
| 57 | orange1.1g042800m | AT4G29680 | 2.238027154 | Alkaline-phosphatase-like family protein |
| 58 | orange1.1g019916m | AT3G14490 | 2.235645785 | Terpenoid cyclases/Protein prenyltransferases superfamily protein |
| 59 | orange1.1g020753m | AT2G33590 | 2.232038955 | NAD(P)-binding Rossmann-fold superfamily protein |
| 60 | orange1.1g041178m | AT4G08850 | 2.227333434 | Leucine-rich repeat receptor-like protein kinase family protein |
| **DEG No** | **DEG**  **(*Citrus sinensis*)** | ***Arabidopsis thaliana* ortholog** | **log_2_ Fold Change** | **Description** |
| 61 | orange1.1g041199m | AT2G04230 | 2.19841544 | FBD, F-box and Leucine Rich Repeat domains containing protein |
| 62 | orange1.1g004582m | AT4G36550 | 2.195825904 | ARM repeat superfamily protein |
| 63 | orange1.1g010494m | AT1G26230 | 2.16282192 | TCP-1/cpn60 chaperonin family protein |
| 64 | orange1.1g028004m | AT5G52020 | 2.127024913 | Integrase-type DNA-binding superfamily protein |
| 65 | orange1.1g028094m | AT5G51990 | 2.119276536 | C-repeat-binding factor 4 |
| 66 | orange1.1g048189m | AT4G31500 | 2.101254024 | cytochrome P450, family 83, subfamily B, polypeptide 1 |
| 67 | orange1.1g014829m | AT2G47460 | 2.093483542 | myb domain protein 12 |
| 68 | orange1.1g035311m | AT1G13980 | 2.084569322 | sec7 domain-containing protein |
| 69 | orange1.1g040647m | AT1G68480 | 2.06204499 | C2H2 and C2HC zinc fingers superfamily protein |
| 70 | orange1.1g000860m | AT2G29940 | 2.01024896 | pleiotropic drug resistance 3 |
| 71 | orange1.1g011487m | AT4G19800 | 2.009876907 | Glycosyl hydrolase family protein with chitinase insertion domain |
| 72 | orange1.1g013951m | AT2G36780 | 2.008835276 | UDP-Glycosyltransferase superfamily protein |
| 73 | orange1.1g002533m | AT5G39000 | 2.006704189 | Malectin/receptor-like protein kinase family protein |
| 74 | orange1.1g046578m | AT5G13730 | 1.985238301 | sigma factor 4 |
| 75 | orange1.1g044114m | AT4G31980 | 1.984183766 | - |
| 76 | orange1.1g042215m | AT5G59030 | 1.975689518 | copper transporter 1 |
| 77 | orange1.1g042997m | AT1G18170 | 1.973610203 | FKBP-like peptidyl-prolyl cis-trans isomerase family protein |
| 78 | orange1.1g025495m | AT4G24380 | 1.934632648 | - |
| 79 | orange1.1g023970m | AT3G01420 | 1.920222108 | Peroxidase superfamily protein |
| 80 | orange1.1g042071m | AT3G08510 | 1.918756833 | phospholipase C 2 |
| 81 | orange1.1g028823m | AT5G67640 | 1.917247253 | - |
| 82 | orange1.1g030470m | AT2G01590 | 1.914888483 | chlororespiratory reduction 3 |
| **DEG No** | **DEG**  **(*Citrus sinensis*)** | ***Arabidopsis thaliana* ortholog** | **log_2_ Fold Change** | **Description** |
| 83 | orange1.1g013717m | AT1G24530 | 1.906167238 | Transducin/WD40 repeat-like superfamily protein |
| 84 | orange1.1g026440m | AT2G03430 | 1.896156608 | Ankyrin repeat family protein |
| 85 | orange1.1g041859m | AT1G16260 | 1.888961102 | Wall-associated kinase family protein |
| 86 | orange1.1g039017m | AT5G65300 | 1.831263509 | - |
| 87 | orange1.1g009058m | AT1G49380 | 1.827749565 | cytochrome c biogenesis protein family |
| 88 | orange1.1g001795m | AT2G24120 | 1.826429411 | DNA/RNA polymerases superfamily protein |
| 89 | orange1.1g029070m | AT1G10200 | 1.821803972 | GATA type zinc finger transcription factor family protein |
| 90 | orange1.1g032375m | AT5G42380 | 1.819404314 | calmodulin like 37 |
| 91 | orange1.1g039142m | AT5G42590 | 1.81459743 | cytochrome P450, family 71, subfamily A, polypeptide 16 |
| 92 | orange1.1g046987m | AT4G24220 | 1.790088183 | NAD(P)-binding Rossmann-fold superfamily protein |
| 93 | orange1.1g023089m | AT5G61440 | 1.747003002 | atypical CYS HIS rich thioredoxin 5 |
| 94 | orange1.1g011426m | AT3G02110 | 1.74031876 | serine carboxypeptidase-like 25 |
| 95 | orange1.1g015832m | AT2G30020 | 1.734972462 | Protein phosphatase 2C family protein |
| 96 | orange1.1g014721m | AT2G23060 | 1.734949918 | Acyl-CoA N-acyltransferases (NAT) superfamily protein |
| 97 | orange1.1g031240m | AT5G22920 | 1.724871249 | CHY-type/CTCHY-type/RING-type Zinc finger protein |
| 98 | orange1.1g031987m | AT4G10300 | 1.719013766 | RmlC-like cupins superfamily protein |
| 99 | orange1.1g009612m | AT5G09970 | 1.686879137 | cytochrome P450, family 78, subfamily A, polypeptide 7 |
| 100 | orange1.1g046355m | AT1G78270 | 1.67251865 | UDP-glucosyl transferase 85A4 |
| 101 | orange1.1g032303m | AT2G32180 | 1.665793366 | plastid transcriptionally active 18 |
| 102 | orange1.1g007834m | AT5G18820 | 1.659596953 | TCP-1/cpn60 chaperonin family protein |
| 103 | orange1.1g041888m | AT5G19650 | 1.657731193 | ovate family protein 8 |
| 104 | orange1.1g022798m | AT2G26660 | 1.654661107 | SPX domain gene 2 |
| **DEG No** | **DEG**  **(*Citrus sinensis*)** | ***Arabidopsis thaliana* ortholog** | **log_2_ Fold Change** | **Description** |
| 105 | orange1.1g047108m | AT3G13960 | 1.647877217 | growth-regulating factor 5 |
| 106 | orange1.1g046829m | AT3G03520 | 1.632982561 | non-specific phospholipase C3 |
| 107 | orange1.1g004956m | AT5G45380 | 1.630793169 | solute:sodium symporters;urea transmembrane transporters |
| 108 | orange1.1g012136m | AT5G67050 | 1.619768997 | alpha/beta-Hydrolases superfamily protein |
| 109 | orange1.1g025173m | AT1G22440 | 1.619323161 | Zinc-binding alcohol dehydrogenase family protein |
| 110 | orange1.1g018336m | AT2G44130 | 1.604632066 | Galactose oxidase/kelch repeat superfamily protein |
| 111 | orange1.1g015736m | AT3G20810 | 1.602198194 | 2-oxoglutarate (2OG) and Fe(II)-dependent oxygenase superfamily protein |
| 112 | orange1.1g011625m | AT1G28050 | 1.599487082 | B-box type zinc finger protein with CCT domain |
| 113 | orange1.1g030975m | AT2G37220 | 1.599280704 | RNA-binding (RRM/RBD/RNP motifs) family protein |
| 114 | orange1.1g029454m | AT5G51160 | 1.594460806 | Ankyrin repeat family protein |
| 115 | orange1.1g004510m | AT3G04580 | 1.565571776 | Signal transduction histidine kinase, hybrid-type, ethylene sensor |
| 116 | orange1.1g016803m | AT3G15850 | 1.558840794 | fatty acid desaturase 5 |
| 117 | orange1.1g005355m | AT1G19390 | 1.548055618 | Wall-associated kinase family protein |
| 118 | orange1.1g043067m | AT3G57230 | 1.539068811 | AGAMOUS-like 16 |
| 119 | orange1.1g032373m | AT5G59770 | 1.527445353 | Protein-tyrosine phosphatase-like, PTPLA |
| 120 | orange1.1g016534m | AT2G15320 | 1.522483463 | Leucine-rich repeat (LRR) family protein |
| 121 | orange1.1g027657m | AT5G24270 | 1.510035634 | Calcium-binding EF-hand family protein |
| 122 | orange1.1g023881m | AT4G11650 | 1.507024584 | osmotin 34 |
| 123 | orange1.1g042503m | AT1G09190 | 1.486084539 | Tetratricopeptide repeat (TPR)-like superfamily protein |
| 124 | orange1.1g029527m | AT1G49720 | 1.476378882 | abscisic acid responsive element-binding factor 1 |
| **DEG No** | **DEG**  **(*Citrus sinensis*)** | ***Arabidopsis thaliana* ortholog** | **log_2_ Fold Change** | **Description** |
| 125 | orange1.1g031330m | AT5G17540 | 1.467670017 | HXXXD-type acyl-transferase family protein |
| 126 | orange1.1g045254m | AT2G36430 | 1.464013342 | Plant protein of unknown function (DUF247) |
| 127 | orange1.1g010656m | AT2G24280 | 1.460050303 | alpha/beta-Hydrolases superfamily protein |
| 128 | orange1.1g041054m | AT2G28840 | 1.45318303 | XB3 ortholog 1 in Arabidopsis thaliana |
| 129 | orange1.1g024682m | AT2G47560 | 1.451630905 | RING/U-box superfamily protein |
| 130 | orange1.1g025356m | AT5G21920 | 1.428517867 | YGGT family protein |
| 131 | orange1.1g028006m | AT5G23960 | 1.422597133 | terpene synthase 21 |
| 132 | orange1.1g034091m | AT1G04900 | 1.41905145 | Protein of unknown function (DUF185) |
| 133 | orange1.1g025772m | AT3G21260 | 1.402713118 | Glycolipid transfer protein (GLTP) family protein |
| 134 | orange1.1g035879m | AT1G17020 | 1.396355389 | senescence-related gene 1 |
| 135 | orange1.1g022200m | AT5G15500 | 1.381392584 | Ankyrin repeat family protein |
| 136 | orange1.1g043149m | AT4G10720 | 1.378535192 | Ankyrin repeat family protein |
| 137 | orange1.1g021201m | AT5G35810 | 1.377408365 | Ankyrin repeat family protein |
| 138 | orange1.1g037537m | AT3G49710 | 1.348493582 | Pentatricopeptide repeat (PPR) superfamily protein |
| 139 | orange1.1g047651m | AT1G78610 | 1.340896199 | mechanosensitive channel of small conductance-like 6 |
| 140 | orange1.1g005508m | AT4G38890 | 1.332306989 | FMN-linked oxidoreductases superfamily protein |
| 141 | orange1.1g012440m | AT1G03940 | 1.328551356 | HXXXD-type acyl-transferase family protein |
| 142 | orange1.1g009759m | AT5G01220 | 1.326947756 | sulfoquinovosyldiacylglycerol 2 |
| 143 | orange1.1g030761m | AT5G56260 | 1.326181613 | Ribonuclease E inhibitor RraA/Dimethylmenaquinone methyltransferase |
| 144 | orange1.1g048155m | AT5G06790 | 1.314972953 | - |
| 145 | orange1.1g018263m | AT3G09010 | 1.297102027 | Protein kinase superfamily protein |
| 146 | orange1.1g031484m | AT1G73740 | 1.290678587 | UDP-Glycosyltransferase superfamily protein |
| 147 | orange1.1g036388m | AT2G29350 | 1.285815396 | senescence-associated gene 13 |
| 148 | orange1.1g025852m | AT1G47740 | 1.285318382 | PPPDE putative thiol peptidase family protein |
| **DEG No** | **DEG**  **(*Citrus sinensis*)** | ***Arabidopsis thaliana* ortholog** | **log_2_ Fold Change** | **Description** |
| 149 | orange1.1g017014m | AT1G55370 | 1.283613472 | NDH-dependent cyclic electron flow 5 |
| 150 | orange1.1g019546m | AT2G40340 | 1.280509051 | Integrase-type DNA-binding superfamily protein |
| 151 | orange1.1g013959m | AT5G65280 | 1.277742473 | GCR2-like 1 |
| 152 | orange1.1g041053m | AT2G31840 | 1.27649487 | Thioredoxin superfamily protein |
| 153 | orange1.1g046676m | AT5G03720 | 1.262725846 | heat shock transcription factor A3 |
| 154 | orange1.1g013592m | AT4G12560 | 1.248637803 | F-box and associated interaction domains-containing protein |
| 155 | orange1.1g014331m | AT4G25500 | 1.231375799 | arginine/serine-rich splicing factor 35 |
| 156 | orange1.1g014565m | AT1G10660 | 1.230900398 | - |
| 157 | orange1.1g030245m | AT2G16600 | 1.221084746 | rotamase CYP 3 |
| 158 | orange1.1g046630m | AT5G57340 | 1.220838528 | - |
| 159 | orange1.1g038097m | AT2G46970 | 1.219908409 | phytochrome interacting factor 3-like 1 |
| 160 | orange1.1g032690m | AT2G47260 | 1.202054755 | WRKY DNA-binding protein 23 |
| 161 | orange1.1g016781m | AT3G12120 | 1.200868658 | fatty acid desaturase 2 |
| 162 | orange1.1g025926m | AT3G52270 | 1.198939209 | Transcription initiation factor IIF, beta subunit |
| 163 | orange1.1g016378m | AT1G67970 | 1.194295779 | heat shock transcription factor A8 |
| 164 | orange1.1g029947m | AT4G14305 | 1.184804158 | Peroxisomal membrane 22 kDa (Mpv17/PMP22) family protein |
| 165 | orange1.1g029508m | AT1G22360 | 1.184048254 | UDP-glucosyl transferase 85A2 |
| 166 | orange1.1g012295m | AT5G23810 | 1.17972236 | amino acid permease 7 |
| 167 | orange1.1g022131m | AT1G52640 | 1.178831712 | Pentatricopeptide repeat (PPR) superfamily protein |
| 168 | orange1.1g030244m | AT5G52780 | 1.172851049 | Protein of unknown function (DUF3464) |
| 169 | orange1.1g047682m | AT1G15520 | 1.169870419 | pleiotropic drug resistance 12 |
| 170 | orange1.1g048578m | AT5G66520 | 1.156746902 | Tetratricopeptide repeat (TPR)-like superfamily protein |
| 171 | orange1.1g024870m | AT1G13700 | 1.154683476 | 6-phosphogluconolactonase 1 |
| 172 | orange1.1g012242m | AT5G39090 | 1.149129312 | HXXXD-type acyl-transferase family protein |
| **DEG No** | **DEG**  **(*Citrus sinensis*)** | ***Arabidopsis thaliana* ortholog** | **log_2_ Fold Change** | **Description** |
| 173 | orange1.1g039105m | AT1G65840 | 1.144357844 | polyamine oxidase 4 |
| 174 | orange1.1g030733m | AT4G36040 | 1.139022683 | Chaperone DnaJ-domain superfamily protein |
| 175 | orange1.1g001088m | AT4G12750 | 1.138856293 | Homeodomain-like transcriptional regulator |
| 176 | orange1.1g033701m | AT1G34640 | 1.13719687 | peptidases |
| 177 | orange1.1g032043m | AT2G03440 | 1.136906881 | nodulin-related protein 1 |
| 178 | orange1.1g041117m | AT5G23530 | 1.133086904 | carboxyesterase 18 |
| 179 | orange1.1g005178m | AT5G46580 | 1.131300251 | pentatricopeptide (PPR) repeat-containing protein |
| 180 | orange1.1g027844m | AT5G48820 | 1.128383256 | inhibitor/interactor with cyclin-dependent kinase |
| 181 | orange1.1g037666m | AT5G04700 | 1.122637929 | Ankyrin repeat family protein |
| 182 | orange1.1g019278m | AT1G71695 | 1.119872339 | Peroxidase superfamily protein |
| 183 | orange1.1g030787m | AT5G14890 | 1.11638887 | NHL domain-containing protein |
| 184 | orange1.1g044451m | AT2G36400 | 1.112809102 | growth-regulating factor 3 |
| 185 | orange1.1g004839m | AT5G66960 | 1.100451081 | Prolyl oligopeptidase family protein |
| 186 | orange1.1g008276m | AT3G49142 | 1.090136744 | Tetratricopeptide repeat (TPR)-like superfamily protein |
| 187 | orange1.1g017936m | AT4G33950 | 1.089644031 | Protein kinase superfamily protein |
| 188 | orange1.1g048204m | AT1G03430 | 1.084398401 | histidine-containing phosphotransfer factor 5 |
| 189 | orange1.1g003020m | AT3G23020 | 1.081084946 | Tetratricopeptide repeat (TPR)-like superfamily protein |
| 190 | orange1.1g017221m | AT4G02340 | 1.076672031 | alpha/beta-Hydrolases superfamily protein |
| 191 | orange1.1g027996m | AT3G15810 | 1.076385165 | Protein of unknown function (DUF567) |
| 192 | orange1.1g040855m | AT4G12330 | 1.073185266 | cytochrome P450, family 706, subfamily A, polypeptide 7 |
| 193 | orange1.1g036421m | AT4G33510 | 1.069820724 | 3-deoxy-d-arabino-heptulosonate 7-phosphate synthase |
| 194 | orange1.1g006076m | AT3G46790 | 1.062907912 | Tetratricopeptide repeat (TPR)-like superfamily protein |
| **DEG No** | **DEG**  **(*Citrus sinensis*)** | ***Arabidopsis thaliana* ortholog** | **log_2_ Fold Change** | **Description** |
| 195 | orange1.1g023918m | AT1G29750 | 1.061555908 | receptor-like kinase in flowers 1 |
| 196 | orange1.1g028071m | AT3G54200 | 1.053809514 | Late embryogenesis abundant (LEA) hydroxyproline-rich glycoprotein family |
| 197 | orange1.1g030085m | AT1G48320 | 1.051989874 | Thioesterase superfamily protein |
| 198 | orange1.1g004565m | AT5G35370 | 1.051496652 | S-locus lectin protein kinase family protein |
| 199 | orange1.1g041521m | AT4G29780 | 1.047127011 | - |
| 200 | orange1.1g044926m | AT5G16370 | 1.03758897 | acyl activating enzyme 5 |
| 201 | orange1.1g012353m | AT4G11820 | 1.029724525 | hydroxymethylglutaryl-CoA synthase / HMG-CoA synthase / 3-hydroxy-3-methylglutaryl coenzyme A synthase |
| 202 | orange1.1g031144m | AT3G48440 | 1.026066623 | Zinc finger C-x8-C-x5-C-x3-H type family protein |
| 203 | orange1.1g039453m | AT2G01730 | 1.024872779 | cleavage and polyadenylation specificity factor 73 kDa subunit-II |
| 204 | orange1.1g037642m | AT3G04300 | 1.005268354 | RmlC-like cupins superfamily protein |
| 205 | orange1.1g043469m | AT1G06460 | 1.000362685 | alpha-crystallin domain 32.1 |
| 206 | orange1.1g032323m | AT2G41430 | 0.998508118 | dehydration-induced protein (ERD15) |
| 207 | orange1.1g006812m | AT2G31240 | 0.99161193 | Tetratricopeptide repeat (TPR)-like superfamily protein |
| 208 | orange1.1g009774m | AT5G53850 | 0.991114645 | haloacid dehalogenase-like hydrolase family protein |
| 209 | orange1.1g021321m | AT4G18370 | 0.987198601 | DEGP protease 5 |
| 210 | orange1.1g026015m | AT5G42900 | 0.986346337 | cold regulated gene 27 |
| 211 | orange1.1g019620m | AT2G39970 | 0.984332049 | Mitochondrial substrate carrier family protein |
| 212 | orange1.1g003725m | AT2G26790 | 0.984221294 | Pentatricopeptide repeat (PPR) superfamily protein |
| 213 | orange1.1g007515m | AT2G24600 | 0.981875831 | Ankyrin repeat family protein |
| 214 | orange1.1g019877m | AT3G18680 | 0.975163386 | Amino acid kinase family protein |
| **DEG No** | **DEG**  **(*Citrus sinensis*)** | ***Arabidopsis thaliana* ortholog** | **log_2_ Fold Change** | **Description** |
| 215 | orange1.1g041942m | AT5G15300 | 0.974618327 | Pentatricopeptide repeat (PPR) superfamily protein |
| 216 | orange1.1g028267m | AT3G09720 | 0.973215904 | P-loop containing nucleoside triphosphate hydrolases superfamily protein |
| 217 | orange1.1g022321m | AT1G22380 | 0.968206818 | UDP-glucosyl transferase 85A3 |
| 218 | orange1.1g001270m | AT1G53390 | 0.96507416 | P-loop containing nucleoside triphosphate hydrolases superfamily protein |
| 219 | orange1.1g031614m | AT5G20230 | 0.963515394 | blue-copper-binding protein |
| 220 | orange1.1g003122m | AT2G41720 | 0.959447891 | Tetratricopeptide repeat (TPR)-like superfamily protein |
| 221 | orange1.1g002019m | AT1G68990 | 0.949271808 | male gametophyte defective 3 |
| 222 | orange1.1g028912m | AT5G43822 | 0.948136572 | Pentatricopeptide repeat (PPR) superfamily protein |
| 223 | orange1.1g033041m | AT3G16720 | 0.946485696 | TOXICOS EN LEVADURA 2 |
| **Down-regulated DEGs** | | | | |
| 1 | orange1.1g042725m | AT5G37540 | -0.946308109 | Eukaryotic aspartyl protease family protein |
| 2 | orange1.1g017274m | AT1G11940 | -0.947144362 | Core-2/I-branching beta-1,6-N-acetylglucosaminyltransferase family protein |
| 3 | orange1.1g021056m | AT3G28480 | -0.957135051 | Oxoglutarate/iron-dependent oxygenase |
| 4 | orange1.1g017010m | AT3G18210 | -0.962601444 | 2-oxoglutarate (2OG) and Fe(II)-dependent oxygenase superfamily protein |
| 5 | orange1.1g047498m | AT1G64430 | -0.973985732 | Pentatricopeptide repeat (PPR) superfamily protein |
| 6 | orange1.1g011821m | AT3G07250 | -0.978537378 | nuclear transport factor 2 (NTF2) family protein / RNA recognition motif (RRM)-containing protein |
| 7 | orange1.1g045147m | AT2G44670 | -0.986988896 | Protein of unknown function (DUF581) |
| 8 | orange1.1g008030m | AT3G23920 | -0.993249212 | beta-amylase 1 |
| **DEG No** | **DEG**  **(*Citrus sinensis*)** | ***Arabidopsis thaliana* ortholog** | **log_2_ Fold Change** | **Description** |
| 9 | orange1.1g005713m | AT5G13650 | -0.9938201 | elongation factor family protein |
| 10 | orange1.1g047348m | AT4G30230 | -1.003702607 | - |
| 11 | orange1.1g014566m | AT4G02920 | -1.006990015 | - |
| 12 | orange1.1g019738m | AT5G42760 | -1.007228119 | Leucine carboxyl methyltransferase |
| 13 | orange1.1g002105m | AT2G25790 | -1.011845644 | Leucine-rich receptor-like protein kinase family protein |
| 14 | orange1.1g014426m | AT5G66460 | -1.015025269 | Glycosyl hydrolase superfamily protein |
| 15 | orange1.1g024141m | AT5G42820 | -1.016321847 | Zinc finger C-x8-C-x5-C-x3-H type family protein |
| 16 | orange1.1g016453m | AT5G20140 | -1.018788433 | SOUL heme-binding family protein |
| 17 | orange1.1g024679m | AT3G14770 | -1.019364715 | Nodulin MtN3 family protein |
| 18 | orange1.1g021845m | AT5G35100 | -1.021113062 | Cyclophilin-like peptidyl-prolyl cis-trans isomerase family protein |
| 19 | orange1.1g008713m | AT5G04190 | -1.029472797 | phytochrome kinase substrate 4 |
| 20 | orange1.1g000432m | AT3G60160 | -1.034935388 | multidrug resistance-associated protein 9 |
| 21 | orange1.1g008950m | AT5G04220 | -1.042528419 | Calcium-dependent lipid-binding (CaLB domain) family protein |
| 22 | orange1.1g034709m | AT1G19020 | -1.053254809 | - |
| 23 | orange1.1g006853m | AT3G14205 | -1.054276168 | Phosphoinositide phosphatase family protein |
| 24 | orange1.1g019224m | AT5G64510 | -1.059563899 | - |
| 25 | orange1.1g048663m | AT1G79270 | -1.059935823 | evolutionarily conserved C-terminal region 8 |
| 26 | orange1.1g045029m | AT3G21760 | -1.066118978 | UDP-Glycosyltransferase superfamily protein |
| 27 | orange1.1g048047m | AT4G20820 | -1.067376777 | FAD-binding Berberine family protein |
| 28 | orange1.1g029411m | AT1G62360 | -1.071666591 | KNOX/ELK homeobox transcription factor |
| 29 | orange1.1g046386m | AT5G45275 | -1.074846433 | Major facilitator superfamily protein |
| 30 | orange1.1g006357m | AT2G32540 | -1.075853965 | cellulose synthase-like B4 |
| 31 | orange1.1g010169m | AT1G71350 | -1.075861257 | eukaryotic translation initiation factor SUI1 family protein |
| **DEG No** | **DEG**  **(*Citrus sinensis*)** | ***Arabidopsis thaliana* ortholog** | **log_2_ Fold Change** | **Description** |
| 32 | orange1.1g017811m | AT3G21640 | -1.101787454 | FKBP-type peptidyl-prolyl cis-trans isomerase family protein |
| 33 | orange1.1g008953m | AT1G59740 | -1.104393737 | Major facilitator superfamily protein |
| 34 | orange1.1g026917m | AT5G35320 | -1.105531234 | - |
| 35 | orange1.1g027827m | AT3G09270 | -1.110980494 | glutathione S-transferase TAU 8 |
| 36 | orange1.1g047612m | AT1G09950 | -1.111913869 | RESPONSE TO ABA AND SALT 1 |
| 37 | orange1.1g005999m | AT5G67200 | -1.114059001 | Leucine-rich repeat protein kinase family protein |
| 38 | orange1.1g002336m | AT1G67720 | -1.115344449 | Leucine-rich repeat protein kinase family protein |
| 39 | orange1.1g036667m | AT3G15620 | -1.11773763 | DNA photolyase family protein |
| 40 | orange1.1g033731m | AT1G12663 | -1.135401254 | - |
| 41 | orange1.1g028963m | AT5G23230 | -1.138453072 | nicotinamidase 2 |
| 42 | orange1.1g032230m | AT1G60950 | -1.145071551 | 2Fe-2S ferredoxin-like superfamily protein |
| 43 | orange1.1g001492m | AT1G04920 | -1.146989678 | sucrose phosphate synthase 3F |
| 44 | orange1.1g041981m | AT4G20050 | -1.155354547 | Pectin lyase-like superfamily protein |
| 45 | orange1.1g022862m | AT5G37660 | -1.155444775 | plasmodesmata-located protein 7 |
| 46 | orange1.1g004829m | AT3G27960 | -1.157863319 | Tetratricopeptide repeat (TPR)-like superfamily protein |
| 47 | orange1.1g042671m | AT4G27830 | -1.165108358 | beta glucosidase 10 |
| 48 | orange1.1g016589m | AT5G11650 | -1.169276092 | alpha/beta-Hydrolases superfamily protein |
| 49 | orange1.1g008986m | AT4G02940 | -1.170628383 | oxidoreductase, 2OG-Fe(II) oxygenase family protein |
| 50 | orange1.1g042442m | AT3G20050 | -1.172416797 | T-complex protein 1 alpha subunit |
| 51 | orange1.1g017798m | AT1G78080 | -1.176523826 | related to AP2 4 |
| 52 | orange1.1g008090m | AT5G60020 | -1.180698363 | laccase 17 |
| 53 | orange1.1g032157m | AT5G05220 | -1.19129485 | - |
| 54 | orange1.1g006141m | AT1G23090 | -1.194840168 | sulfate transporter 91 |
| 55 | orange1.1g010848m | AT2G43330 | -1.197885323 | inositol transporter 1 |
| **DEG No** | **DEG**  **(*Citrus sinensis*)** | ***Arabidopsis thaliana* ortholog** | **log_2_ Fold Change** | **Description** |
| 56 | orange1.1g016506m | AT1G32740 | -1.199017671 | SBP (S-ribonuclease binding protein) family protein |
| 57 | orange1.1g039174m | AT4G36920 | -1.221224743 | Integrase-type DNA-binding superfamily protein |
| 58 | orange1.1g011169m | AT5G39660 | -1.221728289 | cycling DOF factor 2 |
| 59 | orange1.1g034978m | AT4G28290 | -1.23053705 | - |
| 60 | orange1.1g031424m | AT2G16700 | -1.239295172 | actin depolymerizing factor 5 |
| 61 | orange1.1g024728m | AT5G13200 | -1.249539137 | GRAM domain family protein |
| 62 | orange1.1g031575m | AT1G70780 | -1.25070182 | - |
| 63 | orange1.1g004032m | AT5G40390 | -1.250907874 | Raffinose synthase family protein |
| 64 | orange1.1g010581m | AT2G39830 | -1.257638226 | DA1-related protein 2 |
| 65 | orange1.1g022645m | AT1G62400 | -1.259443494 | Protein kinase superfamily protein |
| 66 | orange1.1g048804m | AT4G22160 | -1.260924749 | - |
| 67 | orange1.1g003436m | AT3G46290 | -1.266729225 | hercules receptor kinase 1 |
| 68 | orange1.1g009350m | AT5G44670 | -1.270729984 | Domain of unknown function (DUF23) |
| 69 | orange1.1g016145m | AT1G02630 | -1.276662969 | Nucleoside transporter family protein |
| 70 | orange1.1g006505m | AT2G13610 | -1.279288584 | ABC-2 type transporter family protein |
| 71 | orange1.1g042889m | AT1G67030 | -1.287114868 | zinc finger protein 6 |
| 72 | orange1.1g016540m | AT1G07350 | -1.292518263 | RNA-binding (RRM/RBD/RNP motifs) family protein |
| 73 | orange1.1g046078m | AT2G23090 | -1.292736162 | Uncharacterised protein family SERF |
| 74 | orange1.1g018089m | AT2G15790 | -1.295428439 | peptidyl-prolyl cis-trans isomerase / cyclophilin-40 (CYP40) / rotamase |
| 75 | orange1.1g011057m | AT2G02010 | -1.295440302 | glutamate decarboxylase 4 |
| 76 | orange1.1g043845m | AT3G02468 | -1.298653836 | conserved peptide upstream open reading frame 9 |
| 77 | orange1.1g000272m | AT2G03140 | -1.301126662 | alpha/beta-Hydrolases superfamily protein |
| 78 | orange1.1g025166m | AT5G47740 | -1.304038424 | Adenine nucleotide alpha hydrolases-like superfamily protein |
| **DEG No** | **DEG**  **(*Citrus sinensis*)** | ***Arabidopsis thaliana* ortholog** | **log_2_ Fold Change** | **Description** |
| 79 | orange1.1g031878m | AT4G16447 | -1.307567185 | - |
| 80 | orange1.1g035485m | AT3G18280 | -1.315604381 | Bifunctional inhibitor/lipid-transfer protein/seed storage 2S albumin superfamily protein |
| 81 | orange1.1g020146m | AT5G62020 | -1.318129682 | heat shock transcription factor B2A |
| 82 | orange1.1g031602m | AT1G54050 | -1.32166772 | HSP20-like chaperones superfamily protein |
| 83 | orange1.1g037790m | AT5G60720 | -1.322404594 | Protein of unknown function, DUF547 |
| 84 | orange1.1g020298m | AT3G17611 | -1.330772751 | RHOMBOID-like protein 14 |
| 85 | orange1.1g019411m | AT3G25570 | -1.340708402 | Adenosylmethionine decarboxylase family protein |
| 86 | orange1.1g043241m | AT3G25400 | -1.342673132 | - |
| 87 | orange1.1g033766m | AT3G57810 | -1.34595787 | Cysteine proteinases superfamily protein |
| 88 | orange1.1g000568m | AT3G14470 | -1.358288208 | NB-ARC domain-containing disease resistance protein |
| 89 | orange1.1g043184m | AT5G61890 | -1.359917965 | Integrase-type DNA-binding superfamily protein |
| 90 | orange1.1g029507m | AT5G25190 | -1.36023024 | Integrase-type DNA-binding superfamily protein |
| 91 | orange1.1g039582m | AT3G02470 | -1.360373682 | S-adenosylmethionine decarboxylase |
| 92 | orange1.1g030041m | AT4G27435 | -1.36593957 | Protein of unknown function (DUF1218) |
| 93 | orange1.1g026720m | AT5G45430 | -1.391053308 | Protein kinase superfamily protein |
| 94 | orange1.1g030972m | AT1G15380 | -1.40474285 | Lactoylglutathione lyase / glyoxalase I family protein |
| 95 | orange1.1g013591m | AT5G51760 | -1.407380782 | Protein phosphatase 2C family protein |
| 96 | orange1.1g011048m | AT2G03500 | -1.409110829 | Homeodomain-like superfamily protein |
| 97 | orange1.1g013319m | AT3G08970 | -1.419046739 | DNAJ heat shock N-terminal domain-containing protein |
| 98 | orange1.1g048284m | AT5G59305 | -1.428070623 | - |
| 99 | orange1.1g026954m | AT1G67360 | -1.433123815 | Rubber elongation factor protein (REF) |
| 100 | orange1.1g022135m | AT1G10380 | -1.442723999 | Putative membrane lipoprotein |
| **DEG No** | **DEG**  **(*Citrus sinensis*)** | ***Arabidopsis thaliana* ortholog** | **log_2_ Fold Change** | **Description** |
| 101 | orange1.1g046214m | AT5G10695 | -1.450375236 | - |
| 102 | orange1.1g004659m | AT4G16370 | -1.456227553 | oligopeptide transporter |
| 103 | orange1.1g040625m | AT1G65660 | -1.461575431 | Pre-mRNA splicing Prp18-interacting factor |
| 104 | orange1.1g030911m | AT1G31200 | -1.466405615 | phloem protein 2-A9 |
| 105 | orange1.1g003936m | AT1G69550 | -1.469242027 | disease resistance protein (TIR-NBS-LRR class) |
| 106 | orange1.1g030289m | AT1G10630 | -1.500341563 | ADP-ribosylation factor A1F |
| 107 | orange1.1g012291m | AT1G10740 | -1.507769664 | alpha/beta-Hydrolases superfamily protein |
| 108 | orange1.1g006334m | AT1G18610 | -1.513656719 | Galactose oxidase/kelch repeat superfamily protein |
| 109 | orange1.1g023108m | AT2G37170 | -1.515221942 | plasma membrane intrinsic protein 2 |
| 110 | orange1.1g027685m | AT1G55310 | -1.523375628 | SC35-like splicing factor 33 |
| 111 | orange1.1g026736m | AT1G20450 | -1.529875456 | Dehydrin family protein |
| 112 | orange1.1g040269m | AT5G14410 | -1.53089641 | - |
| 113 | orange1.1g009748m | AT1G60590 | -1.53644971 | Pectin lyase-like superfamily protein |
| 114 | orange1.1g008248m | AT2G23770 | -1.540458971 | protein kinase family protein / peptidoglycan-binding LysM domain-containing protein |
| 115 | orange1.1g023257m | AT3G16050 | -1.548072823 | pyridoxine biosynthesis 1.2 |
| 116 | orange1.1g030233m | AT5G14670 | -1.57538643 | ADP-ribosylation factor A1B |
| 117 | orange1.1g029644m | AT5G54980 | -1.577974816 | Uncharacterised protein family (UPF0497) |
| 118 | orange1.1g035543m | AT1G76240 | -1.587957289 | Arabidopsis protein of unknown function (DUF241) |
| 119 | orange1.1g027186m | AT3G07150 | -1.59587032 | - |
| 120 | orange1.1g024678m | AT5G16010 | -1.596476919 | 3-oxo-5-alpha-steroid 4-dehydrogenase family protein |
| 121 | orange1.1g008233m | AT2G32120 | -1.600402543 | heat-shock protein 70T-2 |
| 122 | orange1.1g041881m | AT1G68190 | -1.615606736 | B-box zinc finger family protein |
| 123 | orange1.1g024199m | AT1G72030 | -1.630258849 | Acyl-CoA N-acyltransferases (NAT) superfamily protein |
| 124 | orange1.1g037884m | AT1G28100 | -1.631568618 | - |
| **DEG No** | **DEG**  **(*Citrus sinensis*)** | ***Arabidopsis thaliana* ortholog** | **log_2_ Fold Change** | **Description** |
| 125 | orange1.1g009192m | AT4G22790 | -1.661912417 | MATE efflux family protein |
| 126 | orange1.1g021653m | AT1G68570 | -1.666120328 | Major facilitator superfamily protein |
| 127 | orange1.1g009392m | AT2G38080 | -1.67363677 | Laccase/Diphenol oxidase family protein |
| 128 | orange1.1g026890m | AT3G56360 | -1.688309156 | - |
| 129 | orange1.1g025108m | AT4G19840 | -1.698283511 | phloem protein 2-A1 |
| 130 | orange1.1g025682m | AT2G42760 | -1.70389443 | - |
| 131 | orange1.1g041158m | AT1G56510 | -1.711780502 | Disease resistance protein (TIR-NBS-LRR class) |
| 132 | orange1.1g048350m | AT4G05320 | -1.728898308 | polyubiquitin 10 |
| 133 | orange1.1g031271m | AT1G59730 | -1.731872215 | thioredoxin H-type 7 |
| 134 | orange1.1g014147m | AT3G02570 | -1.748142941 | Mannose-6-phosphate isomerase, type I |
| 135 | orange1.1g032169m | AT1G21550 | -1.771351006 | Calcium-binding EF-hand family protein |
| 136 | orange1.1g038563m | AT3G10080 | -1.78002256 | RmlC-like cupins superfamily protein |
| 137 | orange1.1g029298m | AT4G25780 | -1.793028874 | CAP (Cysteine-rich secretory proteins, Antigen 5, and Pathogenesis-related 1 protein) superfamily protein |
| 138 | orange1.1g004262m | AT1G01060 | -1.813166231 | Homeodomain-like superfamily protein |
| 139 | orange1.1g014903m | AT5G22060 | -1.81564415 | DNAJ homologue 2 |
| 140 | orange1.1g012245m | AT3G53830 | -1.820049191 | Regulator of chromosome condensation (RCC1) family protein |
| 141 | orange1.1g045080m | AT1G07900 | -1.895278314 | LOB domain-containing protein 1 |
| 142 | orange1.1g021851m | AT1G18330 | -1.902230563 | Homeodomain-like superfamily protein |
| 143 | orange1.1g002728m | AT2G25600 | -1.908096717 | Shaker pollen inward K+ channel |
| 144 | orange1.1g037193m | AT3G55430 | -1.914736602 | O-Glycosyl hydrolases family 17 protein |
| 145 | orange1.1g035679m | AT1G75750 | -1.93562306 | GAST1 protein homolog 1 |
| 146 | orange1.1g031799m | AT3G17130 | -1.971901252 | Plant invertase/pectin methylesterase inhibitor superfamily protein |
| 147 | orange1.1g046009m | AT1G02850 | -1.976122428 | beta glucosidase 11 |
| 148 | orange1.1g033744m | AT5G54940 | -2.008802643 | Translation initiation factor SUI1 family protein |
| **DEG No** | **DEG**  **(*Citrus sinensis*)** | ***Arabidopsis thaliana* ortholog** | **log_2_ Fold Change** | **Description** |
| 149 | orange1.1g029075m | AT2G20560 | -2.015856057 | DNAJ heat shock family protein |
| 150 | orange1.1g005279m | AT1G12240 | -2.021931719 | Glycosyl hydrolases family 32 protein |
| 151 | orange1.1g028463m | AT4G25200 | -2.028168384 | mitochondrion-localized small heat shock protein 23.6 |
| 152 | orange1.1g012057m | AT1G48100 | -2.093096741 | Pectin lyase-like superfamily protein |
| 153 | orange1.1g026833m | AT1G26800 | -2.096870971 | RING/U-box superfamily protein |
| 154 | orange1.1g042221m | AT4G35390 | -2.104786038 | AT-hook protein of GA feedback 1 |
| 155 | orange1.1g048464m | AT1G78860 | -2.121816316 | D-mannose binding lectin protein with Apple-like carbohydrate-binding domain |
| 156 | orange1.1g025415m | AT5G52420 | -2.121965191 | - |
| 157 | orange1.1g018847m | AT5G06720 | -2.152758102 | peroxidase 2 |
| 158 | orange1.1g034330m | AT4G10265 | -2.191470198 | Wound-responsive family protein |
| 159 | orange1.1g032247m | AT4G35580 | -2.210147457 | NAC transcription factor-like 9 |
| 160 | orange1.1g036208m | AT3G12580 | -2.240963143 | heat shock protein 70 |
| 161 | orange1.1g047656m | AT1G02700 | -2.247310271 | - |
| 162 | orange1.1g016497m | AT3G46750 | -2.294677165 | - |
| 163 | orange1.1g020168m | AT5G02500 | -2.307205089 | heat shock cognate protein 70-1 |
| 164 | orange1.1g028228m | AT5G54660 | -2.309451863 | HSP20-like chaperones superfamily protein |
| 165 | orange1.1g046468m | AT1G12060 | -2.322013196 | BCL-2-associated athanogene 5 |
| 166 | orange1.1g048246m | AT4G11660 | -2.338531491 | winged-helix DNA-binding transcription factor family protein |
| 167 | orange1.1g018470m | AT1G48300 | -2.378513628 | - |
| 168 | orange1.1g044398m | AT1G55210 | -2.416468192 | Disease resistance-responsive (dirigent-like protein) family protein |
| 169 | orange1.1g040430m | AT4G16195 | -2.422872148 | Plant self-incompatibility protein S1 family |
| 170 | orange1.1g036950m | AT5G48570 | -2.444647691 | FKBP-type peptidyl-prolyl cis-trans isomerase family protein |
| 171 | orange1.1g045632m | AT4G10310 | -2.534747392 | high-affinity K+ transporter 1 |
| 172 | orange1.1g017694m | AT3G22830 | -2.540250113 | heat shock transcription factor A6B |
| **DEG No** | **DEG**  **(*Citrus sinensis*)** | ***Arabidopsis thaliana* ortholog** | **log_2_ Fold Change** | **Description** |
| 173 | orange1.1g046258m | AT1G74310 | -2.559956309 | heat shock protein 101 |
| 174 | orange1.1g034165m | AT3G21460 | -2.566334833 | Glutaredoxin family protein |
| 175 | orange1.1g020362m | AT5G19040 | -2.604156096 | Isopentenyl transferase 5 |
| 176 | orange1.1g017142m | AT5G22290 | -2.611176332 | NAC domain containing protein 89 |
| 177 | orange1.1g005610m | AT3G44830 | -2.79618693 | Lecithin:cholesterol acyltransferase family protein |
| 178 | orange1.1g005815m | AT5G15250 | -2.798230449 | FTSH protease 6 |
| 179 | orange1.1g039608m | AT1G03020 | -2.798821615 | Thioredoxin superfamily protein |
| 180 | orange1.1g015575m | AT1G12260 | -2.875881046 | NAC 007 |
| 181 | orange1.1g034075m | AT5G18600 | -2.876681459 | Thioredoxin superfamily protein |
| 182 | orange1.1g005370m | AT5G52640 | -3.046602732 | heat shock protein 90.1 |
| 183 | orange1.1g038613m | AT2G37530 | -3.206526001 | - |
| 184 | orange1.1g032067m | AT1G56300 | -3.297053252 | Chaperone DnaJ-domain superfamily protein |
| 185 | orange1.1g031365m | AT1G07400 | -3.336454318 | HSP20-like chaperones superfamily protein |
| 186 | orange1.1g039707m | AT5G17580 | -3.382269598 | Phototropic-responsive NPH3 family protein |
| 187 | orange1.1g016091m | AT2G26150 | -3.72229073 | heat shock transcription factor A2 |

| **Supplemental Table S3.** Differentially expressed genes (DEGs) in stage T_3_ (leaf emergence) of Huanglongbing (HLB)-affected trees compared to healthy trees. | | | | |
| --- | --- | --- | --- | --- |
| **DEG No** | **DEG**  **(*Citrus sinensis*)** | ***Arabidopsis thaliana* ortholog** | **log_2_ Fold Change** | **Description** |
| **Upregulated DEGs** | | | | |
| 1 | orange1.1g021118m | AT5G42180 | 9.138523626 | Peroxidase superfamily protein |
| 2 | orange1.1g035623m | AT4G31500 | 5.688504067 | cytochrome P450, family 83, subfamily B, polypeptide 1 |
| 3 | orange1.1g042543m | AT1G56240 | 5.678939964 | phloem protein 2-B13 |
| 4 | orange1.1g043892m | AT5G42650 | 5.102065836 | allene oxide synthase |
| 5 | orange1.1g010441m | AT2G45220 | 4.854084652 | Plant invertase/pectin methylesterase inhibitor superfamily |
| 6 | orange1.1g019027m | AT4G10490 | 4.787618603 | 2-oxoglutarate (2OG) and Fe(II)-dependent oxygenase superfamily protein |
| 7 | orange1.1g048141m | AT1G17860 | 4.393430337 | Kunitz family trypsin and protease inhibitor protein |
| 8 | orange1.1g044643m | AT1G28710 | 4.393311183 | Nucleotide-diphospho-sugar transferase family protein |
| 9 | orange1.1g045590m | AT1G09155 | 4.334431274 | phloem protein 2-B15 |
| 10 | orange1.1g045328m | AT5G49130 | 4.205862047 | MATE efflux family protein |
| 11 | orange1.1g021203m | AT4G26260 | 4.021916821 | myo-inositol oxygenase 4 |
| 12 | orange1.1g046614m | AT1G29450 | 3.881228899 | SAUR-like auxin-responsive protein family |
| 13 | orange1.1g011801m | AT3G61510 | 3.805923244 | ACC synthase 1 |
| 14 | orange1.1g033054m | AT5G59030 | 3.771140116 | copper transporter 1 |
| 15 | orange1.1g009215m | AT1G66350 | 3.718506516 | RGA-like 1 |
| 16 | orange1.1g038416m | AT5G53970 | 3.528399337 | Tyrosine transaminase family protein |
| 17 | orange1.1g044722m | AT5G06800 | 3.478039281 | myb-like HTH transcriptional regulator family protein |
| 18 | orange1.1g025298m | AT1G20190 | 3.38363031 | expansin 11 |
| 19 | orange1.1g043533m | AT4G35150 | 3.291950692 | O-methyltransferase family protein |
| 20 | orange1.1g016368m | AT1G31350 | 3.249014875 | KAR-UP F-box 1 |
| 21 | orange1.1g041931m | AT3G49190 | 3.217565448 | O-acyltransferase (WSD1-like) family protein |
| **DEG No** | **DEG**  **(*Citrus sinensis*)** | ***Arabidopsis thaliana* ortholog** | **log_2_ Fold Change** | **Description** |
| 22 | orange1.1g037671m | AT5G67140 | 3.208815659 | F-box/RNI-like superfamily protein |
| 23 | orange1.1g021546m | AT1G62420 | 3.206753531 | Protein of unknown function (DUF506) |
| 24 | orange1.1g046195m | AT1G14040 | 3.042548357 | EXS (ERD1/XPR1/SYG1) family protein |
| 25 | orange1.1g039975m | AT4G38840 | 2.975254524 | SAUR-like auxin-responsive protein family |
| 26 | orange1.1g020187m | AT3G12500 | 2.904395222 | basic chitinase |
| 27 | orange1.1g047625m | AT5G66430 | 2.751252551 | S-adenosyl-L-methionine-dependent methyltransferases superfamily protein |
| 28 | orange1.1g014759m | AT1G10970 | 2.721016012 | zinc transporter 4 precursor |
| 29 | orange1.1g043356m | AT2G37030 | 2.70791048 | SAUR-like auxin-responsive protein family |
| 30 | orange1.1g009241m | AT1G12570 | 2.644592832 | Glucose-methanol-choline (GMC) oxidoreductase family protein |
| 31 | orange1.1g037091m | AT2G18950 | 2.607470294 | homogentisate phytyltransferase 1 |
| 32 | orange1.1g026928m | AT3G54420 | 2.556963901 | homolog of carrot EP3-3 chitinase |
| 33 | orange1.1g048132m | AT3G50610 | 2.552274127 | - |
| 34 | orange1.1g035523m | AT3G52790 | 2.535259035 | peptidoglycan-binding LysM domain-containing protein |
| 35 | orange1.1g032389m | AT3G04720 | 2.452377671 | pathogenesis-related 4 |
| 36 | orange1.1g006224m | AT5G49360 | 2.449561589 | beta-xylosidase 1 |
| 37 | orange1.1g043343m | AT4G38770 | 2.434397782 | proline-rich protein 4 |
| 38 | orange1.1g022473m | AT3G63060 | 2.422081543 | EID1-like 3 |
| 39 | orange1.1g018182m | AT3G12750 | 2.395376689 | zinc transporter 1 precursor |
| 40 | orange1.1g016298m | AT1G72220 | 2.363990228 | RING/U-box superfamily protein |
| 41 | orange1.1g027536m | AT1G10460 | 2.343905611 | germin-like protein 7 |
| 42 | orange1.1g017684m | AT5G33370 | 2.189970776 | GDSL-like Lipase/Acylhydrolase superfamily protein |
| 43 | orange1.1g021008m | AT1G56430 | 2.175359615 | nicotianamine synthase 4 |
| 44 | orange1.1g020883m | AT5G51890 | 2.143937147 | Peroxidase superfamily protein |
| 45 | orange1.1g025347m | AT2G40610 | 2.124933504 | expansin A8 |
| **DEG No** | **DEG**  **(*Citrus sinensis*)** | ***Arabidopsis thaliana* ortholog** | **log_2_ Fold Change** | **Description** |
| 46 | orange1.1g046361m | AT2G34790 | 2.118633539 | FAD-binding Berberine family protein |
| 47 | orange1.1g028728m | AT1G26650 | 2.111198968 | - |
| 48 | orange1.1g007932m | AT2G40460 | 2.097118041 | Major facilitator superfamily protein |
| 49 | orange1.1g039472m | AT4G25040 | 2.09405724 | Uncharacterised protein family (UPF0497) |
| 50 | orange1.1g017143m | AT4G17370 | 2.09200778 | Oxidoreductase family protein |
| 51 | orange1.1g011487m | AT4G19800 | 2.08115184 | Glycosyl hydrolase family protein with chitinase insertion domain |
| 52 | orange1.1g009018m | AT2G42560 | 2.067731777 | late embryogenesis abundant domain-containing protein / LEA domain-containing protein |
| 53 | orange1.1g033544m | AT2G38540 | 2.066182697 | lipid transfer protein 1 |
| 54 | orange1.1g032805m | AT1G54120 | 2.059723313 | - |
| 55 | orange1.1g029877m | AT5G25840 | 2.036744856 | Protein of unknown function (DUF1677) |
| 56 | orange1.1g038598m | AT5G60020 | 2.014538561 | laccase 17 |
| 57 | orange1.1g021017m | AT5G67210 | 2.006071713 | Protein of unknown function (DUF579) |
| 58 | orange1.1g046180m | AT5G38760 | 1.996244311 | Late embryogenesis abundant protein (LEA) family protein |
| 59 | orange1.1g044848m | AT3G26040 | 1.991687749 | HXXXD-type acyl-transferase family protein |
| 60 | orange1.1g008877m | AT3G18440 | 1.986977378 | aluminum-activated malate transporter 9 |
| 61 | orange1.1g036528m | AT5G46940 | 1.985515067 | Plant invertase/pectin methylesterase inhibitor superfamily protein |
| 62 | orange1.1g026047m | AT5G03170 | 1.969511559 | FASCICLIN-like arabinogalactan-protein 11 |
| 63 | orange1.1g043643m | AT5G60710 | 1.968510478 | Zinc finger (C3HC4-type RING finger) family protein |
| 64 | orange1.1g024380m | AT5G03140 | 1.963867935 | Concanavalin A-like lectin protein kinase family protein |
| 65 | orange1.1g011311m | AT1G07250 | 1.96211383 | UDP-glucosyl transferase 71C4 |
| 66 | orange1.1g023384m | AT4G35160 | 1.940161129 | O-methyltransferase family protein |
| 67 | orange1.1g015195m | AT4G37680 | 1.924839366 | heptahelical protein 4 |
| 68 | orange1.1g009887m | AT4G12320 | 1.922652764 | cytochrome P450, family 706, subfamily A, polypeptide 6 |
| 69 | orange1.1g029634m | AT3G16920 | 1.891010364 | chitinase-like protein 2 |
| **DEG No** | **DEG**  **(*Citrus sinensis*)** | ***Arabidopsis thaliana* ortholog** | **log_2_ Fold Change** | **Description** |
| 70 | orange1.1g007610m | AT1G05590 | 1.884212657 | beta-hexosaminidase 2 |
| 71 | orange1.1g035968m | AT1G01490 | 1.871227379 | Heavy metal transport/detoxification superfamily protein |
| 72 | orange1.1g025320m | AT1G07050 | 1.862002371 | CCT motif family protein |
| 73 | orange1.1g012100m | AT1G12740 | 1.829479596 | cytochrome P450, family 87, subfamily A, polypeptide 2 |
| 74 | orange1.1g008315m | AT4G33790 | 1.820720545 | Jojoba acyl CoA reductase-related male sterility protein |
| 75 | orange1.1g039429m | AT5G41761 | 1.809219191 | - |
| 76 | orange1.1g008732m | AT1G64940 | 1.797853314 | cytochrome P450, family 87, subfamily A, polypeptide 6 |
| 77 | orange1.1g034697m | AT1G75250 | 1.792210662 | RAD-like 6 |
| 78 | orange1.1g013672m | AT2G03200 | 1.786364794 | Eukaryotic aspartyl protease family protein |
| 79 | orange1.1g027725m | AT3G62020 | 1.772211241 | germin-like protein 10 |
| 80 | orange1.1g009835m | AT2G34930 | 1.750382428 | disease resistance family protein / LRR family protein |
| 81 | orange1.1g030750m | AT1G43790 | 1.749759266 | tracheary element differentiation-related 6 |
| 82 | orange1.1g010101m | AT3G06350 | 1.744931294 | dehydroquinate dehydratase, putative / shikimate dehydrogenase, putative |
| 83 | orange1.1g021577m | AT2G41610 | 1.741532734 | - |
| 84 | orange1.1g012895m | AT3G52820 | 1.734311465 | purple acid phosphatase 22 |
| 85 | orange1.1g018195m | AT5G45950 | 1.728497179 | GDSL-like Lipase/Acylhydrolase superfamily protein |
| 86 | orange1.1g043166m | AT1G09610 | 1.722366987 | Protein of unknown function (DUF579) |
| 87 | orange1.1g009844m | AT4G31940 | 1.711683465 | cytochrome P450, family 82, subfamily C, polypeptide 4 |
| 88 | orange1.1g000475m | AT1G04120 | 1.688571138 | multidrug resistance-associated protein 5 |
| 89 | orange1.1g023044m | AT1G77160 | 1.686558646 | Protein of unknown function (DUF506) |
| 90 | orange1.1g009805m | AT4G03500 | 1.683779408 | Ankyrin repeat family protein |
| 91 | orange1.1g019020m | AT5G15780 | 1.665054545 | Pollen Ole e 1 allergen and extensin family protein |
| 92 | orange1.1g023506m | AT1G03980 | 1.664311388 | phytochelatin synthase 2 |
| 93 | orange1.1g014457m | AT5G65890 | 1.660695534 | ACT domain repeat 1 |
| **DEG No** | **DEG**  **(*Citrus sinensis*)** | ***Arabidopsis thaliana* ortholog** | **log_2_ Fold Change** | **Description** |
| 94 | orange1.1g042552m | AT2G28315 | 1.643514518 | Nucleotide/sugar transporter family protein |
| 95 | orange1.1g007291m | AT1G30100 | 1.637239023 | nine-cis-epoxycarotenoid dioxygenase 5 |
| 96 | orange1.1g006929m | AT4G29210 | 1.628795574 | gamma-glutamyl transpeptidase 4 |
| 97 | orange1.1g045034m | AT4G39340 | 1.614987289 | Protein of unknown function (DUF1278) |
| 98 | orange1.1g028208m | AT3G44220 | 1.611119643 | Late embryogenesis abundant (LEA) hydroxyproline-rich glycoprotein family |
| 99 | orange1.1g041212m | AT2G35150 | 1.599099095 | EXORDIUM like 1 |
| 100 | orange1.1g012090m | AT1G68470 | 1.597452248 | Exostosin family protein |
| 101 | orange1.1g043646m | AT1G08490 | 1.597192715 | chloroplastic NIFS-like cysteine desulfurase |
| 102 | orange1.1g011040m | AT2G45550 | 1.595965281 | cytochrome P450, family 76, subfamily C, polypeptide 4 |
| 103 | orange1.1g040825m | AT5G16490 | 1.58847504 | ROP-interactive CRIB motif-containing protein 4 |
| 104 | orange1.1g041859m | AT1G16260 | 1.563344183 | Wall-associated kinase family protein |
| 105 | orange1.1g036972m | AT5G17540 | 1.558503177 | HXXXD-type acyl-transferase family protein |
| 106 | orange1.1g045358m | AT4G01850 | 1.548808814 | S-adenosylmethionine synthetase 2 |
| 107 | orange1.1g016039m | AT2G37040 | 1.534901824 | PHE ammonia lyase 1 |
| 108 | orange1.1g037064m | AT4G34350 | 1.527788435 | 4-hydroxy-3-methylbut-2-enyl diphosphate reductase |
| 109 | orange1.1g038785m | AT3G19000 | 1.524399764 | 2-oxoglutarate (2OG) and Fe(II)-dependent oxygenase superfamily protein |
| 110 | orange1.1g016580m | AT4G08300 | 1.524299571 | nodulin MtN21 /EamA-like transporter family protein |
| 111 | orange1.1g017485m | AT5G61520 | 1.522222525 | Major facilitator superfamily protein |
| 112 | orange1.1g001574m | AT5G44030 | 1.521508178 | cellulose synthase A4 |
| 113 | orange1.1g042053m | AT3G48290 | 1.497968344 | cytochrome P450, family 71, subfamily A, polypeptide 24 |
| 114 | orange1.1g014084m | AT5G01360 | 1.47896828 | Plant protein of unknown function (DUF828) |
| 115 | orange1.1g044843m | AT5G62180 | 1.472650518 | S-adenosylmethionine synthetase 2 |
| 116 | orange1.1g018590m | AT2G04570 | 1.461309666 | GDSL-like Lipase/Acylhydrolase superfamily protein |
| 117 | orange1.1g039060m | AT5G17420 | 1.45841073 | Cellulose synthase family protein |
| **DEG No** | **DEG**  **(*Citrus sinensis*)** | ***Arabidopsis thaliana* ortholog** | **log_2_ Fold Change** | **Description** |
| 118 | orange1.1g009917m | AT1G19250 | 1.457166797 | flavin-dependent monooxygenase 1 |
| 119 | orange1.1g048184m | AT5G07990 | 1.456670394 | Cytochrome P450 superfamily protein |
| 120 | orange1.1g042461m | AT3G29635 | 1.45546661 | HXXXD-type acyl-transferase family protein |
| 121 | orange1.1g017946m | AT3G51240 | 1.442442848 | flavanone 3-hydroxylase |
| 122 | orange1.1g027258m | AT1G26820 | 1.440909318 | ribonuclease 3 |
| 123 | orange1.1g015342m | AT5G09220 | 1.434116975 | amino acid permease 2 |
| 124 | orange1.1g033495m | AT1G74670 | 1.428745335 | Gibberellin-regulated family protein |
| 125 | orange1.1g009292m | AT2G46660 | 1.427382963 | cytochrome P450, family 78, subfamily A, polypeptide 6 |
| 126 | orange1.1g041217m | AT4G19810 | 1.421461336 | Glycosyl hydrolase family protein with chitinase insertion domain |
| 127 | orange1.1g039669m | AT1G68620 | 1.416435794 | alpha/beta-Hydrolases superfamily protein |
| 128 | orange1.1g008331m | AT5G51950 | 1.389975768 | Glucose-methanol-choline (GMC) oxidoreductase family protein |
| 129 | orange1.1g039456m | AT4G33980 | 1.379289558 | - |
| 130 | orange1.1g036436m | AT3G16520 | 1.370200848 | UDP-glucosyl transferase 88A1 |
| 131 | orange1.1g047228m | AT5G20410 | 1.368820974 | monogalactosyldiacylglycerol synthase 2 |
| 132 | orange1.1g037797m | AT4G33550 | 1.364134147 | Bifunctional inhibitor/lipid-transfer protein/seed storage 2S albumin superfamily protein |
| 133 | orange1.1g015500m | AT2G32510 | 1.36366555 | mitogen-activated protein kinase kinase kinase 17 |
| 134 | orange1.1g038146m | AT1G28400 | 1.363657527 | - |
| 135 | orange1.1g011580m | AT1G77380 | 1.363493492 | amino acid permease 3 |
| 136 | orange1.1g003897m | AT5G40390 | 1.347541187 | Raffinose synthase family protein |
| 137 | orange1.1g041419m | AT3G50740 | 1.334937478 | UDP-glucosyl transferase 72E1 |
| 138 | orange1.1g013326m | AT2G39890 | 1.334176273 | proline transporter 1 |
| 139 | orange1.1g043241m | AT3G25400 | 1.325438526 | - |
| 140 | orange1.1g021737m | AT1G75280 | 1.312388866 | NmrA-like negative transcriptional regulator family protein |
| 141 | orange1.1g011935m | AT5G17740 | 1.312035742 | P-loop containing nucleoside triphosphate hydrolases superfamily protein |
| **DEG No** | **DEG**  **(*Citrus sinensis*)** | ***Arabidopsis thaliana* ortholog** | **log_2_ Fold Change** | **Description** |
| 142 | orange1.1g046375m | AT5G54160 | 1.288288453 | O-methyltransferase 1 |
| 143 | orange1.1g043506m | AT3G48180 | 1.272043052 | - |
| 144 | orange1.1g035460m | AT3G05890 | 1.271670367 | Low temperature and salt responsive protein family |
| 145 | orange1.1g024728m | AT5G13200 | 1.267475318 | GRAM domain family protein |
| 146 | orange1.1g047310m | AT1G13710 | 1.259972663 | cytochrome P450, family 78, subfamily A, polypeptide 5 |
| 147 | orange1.1g032228m | AT2G04520 | 1.250385709 | Nucleic acid-binding, OB-fold-like protein |
| 148 | orange1.1g030729m | AT3G27200 | 1.249761322 | Cupredoxin superfamily protein |
| 149 | orange1.1g019105m | AT5G61340 | 1.245474008 | - |
| 150 | orange1.1g029380m | AT5G62200 | 1.240507495 | Embryo-specific protein 3, (ATS3) |
| 151 | orange1.1g014836m | AT2G40320 | 1.236041102 | TRICHOME BIREFRINGENCE-LIKE 33 |
| 152 | orange1.1g039426m | AT4G16820 | 1.220935663 | alpha/beta-Hydrolases superfamily protein |
| 153 | orange1.1g025740m | AT1G69530 | 1.210702932 | expansin A1 |
| 154 | orange1.1g044312m | AT1G79680 | 1.204018236 | WALL ASSOCIATED KINASE (WAK)-LIKE 10 |
| 155 | orange1.1g028110m | AT1G05000 | 1.199074713 | Phosphotyrosine protein phosphatases superfamily protein |
| 156 | orange1.1g016679m | AT3G48460 | 1.19170614 | GDSL-like Lipase/Acylhydrolase superfamily protein |
| 157 | orange1.1g032043m | AT2G03440 | 1.184612893 | nodulin-related protein 1 |
| 158 | orange1.1g040271m | AT3G50170 | 1.154878403 | Plant protein of unknown function (DUF247) |
| 159 | orange1.1g025959m | AT2G20520 | 1.15293112 | FASCICLIN-like arabinogalactan 6 |
| 160 | orange1.1g002672m | AT3G45140 | 1.152572065 | lipoxygenase 2 |
| 161 | orange1.1g008374m | AT5G06740 | 1.15105167 | Concanavalin A-like lectin protein kinase family protein |
| 162 | orange1.1g039041m | AT3G48770 | 1.146122456 | DNA binding;ATP binding |
| 163 | orange1.1g034453m | AT5G43150 | 1.145744623 | - |
| 164 | orange1.1g011743m | AT1G27040 | 1.112344426 | Major facilitator superfamily protein |
| 165 | orange1.1g018567m | AT3G50760 | 1.107005344 | galacturonosyltransferase-like 2 |
| 166 | orange1.1g023148m | AT2G35700 | 1.088240457 | ERF family protein 38 |
| **DEG No** | **DEG**  **(*Citrus sinensis*)** | ***Arabidopsis thaliana* ortholog** | **log_2_ Fold Change** | **Description** |
| 167 | orange1.1g031044m | AT5G61660 | 1.083754538 | glycine-rich protein |
| 168 | orange1.1g001705m | AT4G10120 | 1.083427994 | Sucrose-phosphate synthase family protein |
| 169 | orange1.1g040187m | AT5G23530 | 1.083426079 | carboxyesterase 18 |
| 170 | orange1.1g013322m | AT1G61050 | 1.078681816 | alpha 1,4-glycosyltransferase family protein |
| 171 | orange1.1g018906m | AT2G38110 | 1.056796245 | glycerol-3-phosphate acyltransferase 6 |
| 172 | orange1.1g001384m | AT1G74360 | 1.054476382 | Leucine-rich repeat protein kinase family protein |
| 173 | orange1.1g010334m | AT3G26300 | 1.051127151 | cytochrome P450, family 71, subfamily B, polypeptide 34 |
| 174 | orange1.1g034709m | AT1G19020 | 1.05075662 | - |
| 175 | orange1.1g009363m | AT1G47670 | 1.037939484 | Transmembrane amino acid transporter family protein |
| 176 | orange1.1g014192m | AT5G15900 | 1.035972391 | TRICHOME BIREFRINGENCE-LIKE 19 |
| 177 | orange1.1g029298m | AT4G25780 | 1.034362705 | CAP (Cysteine-rich secretory proteins, Antigen 5, and Pathogenesis-related 1 protein) superfamily protein |
| 178 | orange1.1g021921m | AT4G33180 | 1.032448538 | alpha/beta-Hydrolases superfamily protein |
| 179 | orange1.1g016490m | AT3G02630 | 1.010846906 | Plant stearoyl-acyl-carrier-protein desaturase family protein |
| 180 | orange1.1g012057m | AT1G48100 | 1.008965502 | Pectin lyase-like superfamily protein |
| 181 | orange1.1g045267m | AT4G01070 | 0.997832288 | UDP-Glycosyltransferase superfamily protein |
| 182 | orange1.1g040903m | AT1G78110 | 0.985515884 | - |
| 183 | orange1.1g037704m | AT5G55340 | 0.984069952 | MBOAT (membrane bound O-acyl transferase) family protein |
| 184 | orange1.1g016380m | AT5G06750 | 0.970302748 | Protein phosphatase 2C family protein |
| 185 | orange1.1g043704m | AT4G15440 | 0.958344897 | hydroperoxide lyase 1 |
| 186 | orange1.1g003980m | AT3G19620 | 0.954260405 | Glycosyl hydrolase family protein |
| 187 | orange1.1g009728m | AT1G22410 | 0.952160623 | Class-II DAHP synthetase family protein |
| 188 | orange1.1g007760m | AT4G17220 | 0.947626387 | microtubule-associated proteins 70-5 |
| **DEG No** | **DEG**  **(*Citrus sinensis*)** | ***Arabidopsis thaliana* ortholog** | **log_2_ Fold Change** | **Description** |
| **Down-regulated DEGs** | | | | |
| 1 | orange1.1g045795m | AT1G62780 | -0.97368616 | - |
| 2 | orange1.1g037286m | AT5G27390 | -1.022039189 | Mog1/PsbP/DUF1795-like photosystem II reaction center PsbP family protein |
| 3 | orange1.1g031837m | AT1G08830 | -1.034232894 | copper/zinc superoxide dismutase 1 |
| 4 | orange1.1g026085m | AT1G74240 | -1.040918058 | Mitochondrial substrate carrier family protein |
| 5 | orange1.1g020927m | AT4G23420 | -1.068650846 | NAD(P)-binding Rossmann-fold superfamily protein |
| 6 | orange1.1g023089m | AT5G61440 | -1.095324261 | atypical CYS HIS rich thioredoxin 5 |
| 7 | orange1.1g010604m | AT5G55580 | -1.099656861 | Mitochondrial transcription termination factor family protein |
| 8 | orange1.1g040633m | AT5G19030 | -1.153640403 | RNA-binding (RRM/RBD/RNP motifs) family protein |
| 9 | orange1.1g000230m | AT4G28080 | -1.19399174 | Tetratricopeptide repeat (TPR)-like superfamily protein |
| 10 | orange1.1g037904m | AT5G45200 | -1.47830476 | Disease resistance protein (TIR-NBS-LRR class) family |
| 11 | orange1.1g013515m | AT5G26180 | -1.538188602 | S-adenosyl-L-methionine-dependent methyltransferases superfamily protein |
| 12 | orange1.1g011458m | AT5G39940 | -1.553281225 | S-adenosyl-L-methionine-dependent methyltransferases superfamily protein |
| 13 | orange1.1g041396m | AT4G39800 | -2.924785634 | myo-inositol-1-phosphate synthase 1 |


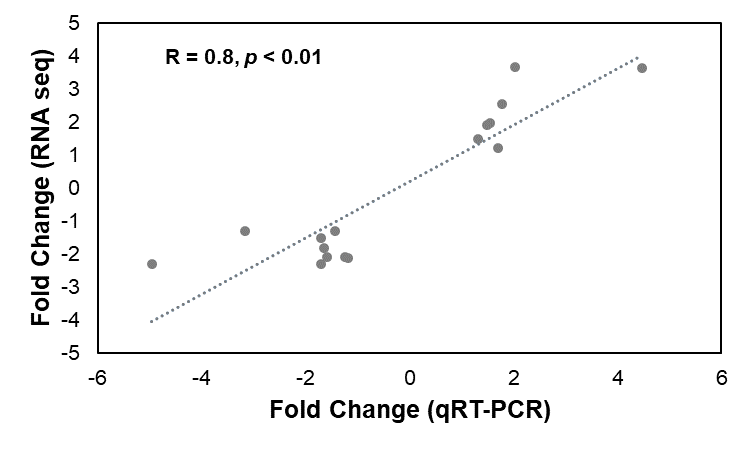


**Supplemental Figure S1:** Pearson Correlation between relative fold changes in expression based on quantitative real-time polymerase chain reaction (qRT-PCR) and RNA-seq.
